# A systematic review of the asymmetric inheritance of cellular organelles in eukaryotes: A critique of basic science validity and imprecision

**DOI:** 10.1371/journal.pone.0178645

**Published:** 2017-05-31

**Authors:** Anne Collins, Janine Ross, Shona H. Lang

**Affiliations:** 1 Department of Biology, University of York, Heslington, United Kingdom; 2 Independent, York, United Kingdom; Institut de Genetique et Developpement de Rennes, FRANCE

## Abstract

We performed a systematic review to identify all original publications describing the asymmetric inheritance of cellular organelles in normal animal eukaryotic cells and to critique the validity and imprecision of the evidence. Searches were performed in Embase, MEDLINE and Pubmed up to November 2015. Screening of titles, abstracts and full papers was performed by two independent reviewers. Data extraction and validity were performed by one reviewer and checked by a second reviewer. Study quality was assessed using the SYRCLE risk of bias tool, for animal studies and by developing validity tools for the experimental model, organelle markers and imprecision. A narrative data synthesis was performed. We identified 31 studies (34 publications) of the asymmetric inheritance of organelles after mitotic or meiotic division. Studies for the asymmetric inheritance of centrosomes (n = 9); endosomes (n = 6), P granules (n = 4), the midbody (n = 3), mitochondria (n = 3), proteosomes (n = 2), spectrosomes (n = 2), cilia (n = 2) and endoplasmic reticulum (n = 2) were identified. Asymmetry was defined and quantified by variable methods. Assessment of the statistical reliability of the results indicated only two studies (7%) were judged to have low concern, the majority of studies (77%) were 'unclear' and five (16%) were judged to have 'high concerns'; the main reasons were low technical repeats (<10). Assessment of model validity indicated that the majority of studies (61%) were judged to be valid, ten studies (32%) were unclear and two studies (7%) were judged to have 'high concerns'; both described 'stem cells' without providing experimental evidence to confirm this (pluripotency and self-renewal). Assessment of marker validity indicated that no studies had low concern, most studies were unclear (96.5%), indicating there were insufficient details to judge if the markers were appropriate. One study had high concern for marker validity due to the contradictory results of two markers for the same organelle. For most studies the validity and imprecision of results could not be confirmed. In particular, data were limited due to a lack of reporting of interassay variability, sample size calculations, controls and functional validation of organelle markers. An evaluation of 16 systematic reviews containing cell assays found that only 50% reported adherence to PRISMA or ARRIVE reporting guidelines and 38% reported a formal risk of bias assessment. 44% of the reviews did not consider how relevant or valid the models were to the research question. 75% reviews did not consider how valid the markers were. 69% of reviews did not consider the impact of the statistical reliability of the results. Future systematic reviews in basic or preclinical research should ensure the rigorous reporting of the statistical reliability of the results in addition to the validity of the methods. Increased awareness of the importance of reporting guidelines and validation tools is needed for the scientific community.

## Introduction

A systematic review uses transparent and systematic methods to minimise bias and random error. It seeks to combine the results from multiple studies to generate a more powerful, overall finding about the effectiveness of a technology or evidence under investigation. Systematic reviews have been used predominantly for clinical research, but it is possible to conduct a review in any field, or in any topic, by synthesising different types of evidence. Systematic review techniques are rigorous, to minimise bias, and include a critical appraisal of the quality of the evidence. Traditional reviews often include a range of sources of potential bias including: failure to clearly state the review question, no attempt to identify all relevant literature, poor explanation of the inclusion and exclusion of evidence, lack of consideration of study methods or quality, and typically are conducted by a single reviewer with a professional interest in the field [1]. Bias is a systematic error, usually due to an issue with study design, such that repetition of the same experiment would still give the wrong answer. Imprecision is the random error, repetition of the result will reduce sampling error, therefore larger studies are more precise.

The use of systematic reviews within basic research is not commonly performed. In recent years there have been systematic reviews of preclinical, animal-based studies. These reviews have led to the establishment of CAMARADES (Collaborative Approach to Meta-Analysis and Review of Animal Data from Experimental Studies; http://www.dcn.ed.ac.uk/camarades/default.htm), the development of the ARRIVE reporting guidelines for in vivo studies [2], and the SYRCLE risk of bias tool [3]. Systematic reviews for basic science offer the same advantages as for those carried out for preclinical animal studies: to statistically combine the results of a number of similar studies, to provide more reliable results upon which to base decisions (scientific rather than subjective), to identify evidence gaps, to allow an evidence based translation of basic science to the clinic, and to improve the validation of basic research by identifying results within multiple model systems [4].

The authors of this review have published on the fate and differentiation of human prostate stem cells [5–8], as well as performing clinical systematic reviews [9–12]. Stem cell research is of great biomedical interest and stem cell therapies are a growing area of research, although the basic biology is not well understood. Whilst performing cell tracking experiments, we observed the asymmetric inheritance of a fluorescent lipid soluble dye known as PKH26 [5,6]. Many groups have observed that PKH26 becomes internalised into the endocytic pathway [13], which led us to postulate that we had witnessed early asymmetric inheritance of lipid membrane organelles. We decided to perform a systematic review to establish what evidence exists for the asymmetric inheritance of any eukaryotic organelle after cell division, and specifically to identify if this can occur in stem cells or human cells. We discovered that existing risk of bias tools were not appropriate for cell based studies. Therefore, we produced validity tools to assess: a) the model, b) the marker and c) the imprecision of the result. This review concentrates on the assessment of the scientific quality of the included studies (rather than the findings). We aimed to demonstrate the validity and reliability of the included studies and we aimed to evaluate whether other basic science systematic reviews assessed quality or experimental validity and imprecision.

## Methods

The methods for the literature searches and systematic review adhered to the Cochrane Collaboration guidance [14], to reduce the risk of bias and error. This study was reported according to the Preferred Reporting Items for Systematic Reviews and Meta-Analyses (PRISMA) statement [15]. The study protocol was registered with the Collaborative Approach to Meta Analysis and Review of Animal Data from Experimental Studies (CAMARADES), http://www.dcn.ed.ac.uk/camarades/default.htm.

### Definitions

Asymmetric inheritance was defined as the division of an organelle and its inheritance to either the mother or daughter cell but not to both, or the unequal inheritance between two daughter cells.

### Literature searches

Attempts were made to identify studies on asymmetric cell division. Searches in bibliographic databases were not limited by publication date, language or publication status (published or unpublished). Search strategies are presented in S1 File. The following databases were searched on 18 November 2015: Embase (OvidSP): 1974–2015/11/18, Medline (OvidSP): 1946–2015/11/WK2, Medline In-Process Citations & Daily Update (OvidSP): up to 2015/11/18, PubMed (NLM) (Internet) (http://www.ncbi.nlm.nih.gov/pubmed): up to 2015/11/19. The reference lists of all included articles and relevant reviews were also searched to identify studies for inclusion. A second search was performed to identify systematic reviews of basic research which included eukaryotic and cell based assays; the methods used are described above.

### Inclusion and exclusion criteria

We included original publications of the asymmetric division (mitotic or meiotic) or inheritance of cellular organelles in normal animal eukaryotic cells. We specifically included the following organelles: golgi, endoplasmic reticulum, sarcoplastic reticulum, mitochondria, vacuole, proteosome, lysosome, centrosome, microtubule organising centre, centriole, spindle pole body, autophagosome, exosome, peroxisome. The inclusion criteria are summarised in S1 Table. Other organelles were considered if identified from the searches.

We excluded prokaryotes, plants and models derived from diseased eukaryotic animals (e.g. cancer cell lines). Non-English language articles, conference proceedings, abstracts, commentaries and reviews were not included. Studies which examined intracellular polar distribution or asymmetric localisation in the absence of cell division were not included. Proteins, genes, transcription factors, cell size, chromosomes, chromatids were not included.

The second review included any systematic reviews of basic research which included cell based assays. We excluded all reviews which included clinical, diagnostic or prognostics outcomes (S1 Table).

### Study selection and data extraction

Publications were loaded onto the systematic review web app, Rayyan, for title and abstract screening [16]. Titles and abstracts were independently screened by two reviewers. Articles meeting the inclusion criteria were obtained as full paper copies. These were independently examined in detail by two reviewers to determine whether the full paper met the inclusion criteria of the review. All papers excluded at this second stage of the screening process were documented along with the reasons for exclusion. Any discrepancies between reviewers were resolved through consensus.

Data extraction was performed by one reviewer and checked by a second reviewer. Any discrepancies were resolved through discussion. Studies were identified by the surname of the first author and by the publication year. To avoid the duplication of data, multiple publications from the same research group were not extracted (if reporting the same methodology for the same marker and model, and reporting the same result for asymmetry but the authors were progressing the research), instead the paper providing the most robust evidence (imprecision or marker validity) was chosen as the primary evidence source. When a study identified more than one marker for a given organelle, all results for all markers were extracted (even if one marker did not identify asymmetric inheritance).

### Quality assessment

Study quality was initially assessed using SYRCLE's risk of bias tool for animal studies [3]. Further validity assessments were created based on the requirements of the authors and by considering the ARRIVE Guidelines Checklist [2], the ROBINS-I tools [17], Downs and Black checklist [18], and QUADAS-2 [19]. These new validity tools were based on model validity, statistical imprecision and marker/experimental validity (S2–S4 Tables). Two reviewers independently assessed study quality and any discrepancies were resolved through discussion.

### Data synthesis

A narrative summary of all the included studies was compiled. The data were sorted according to organelle and according to phylogenic order. The organisms most direct to humans were ordered to the top of the table and organisms most indirect to the bottom. Results were presented alongside overall judgements for concerns regarding the validity and imprecision of the result.

## Results and discussion

### Literature searches and inclusion assessment

**Fig 1:**
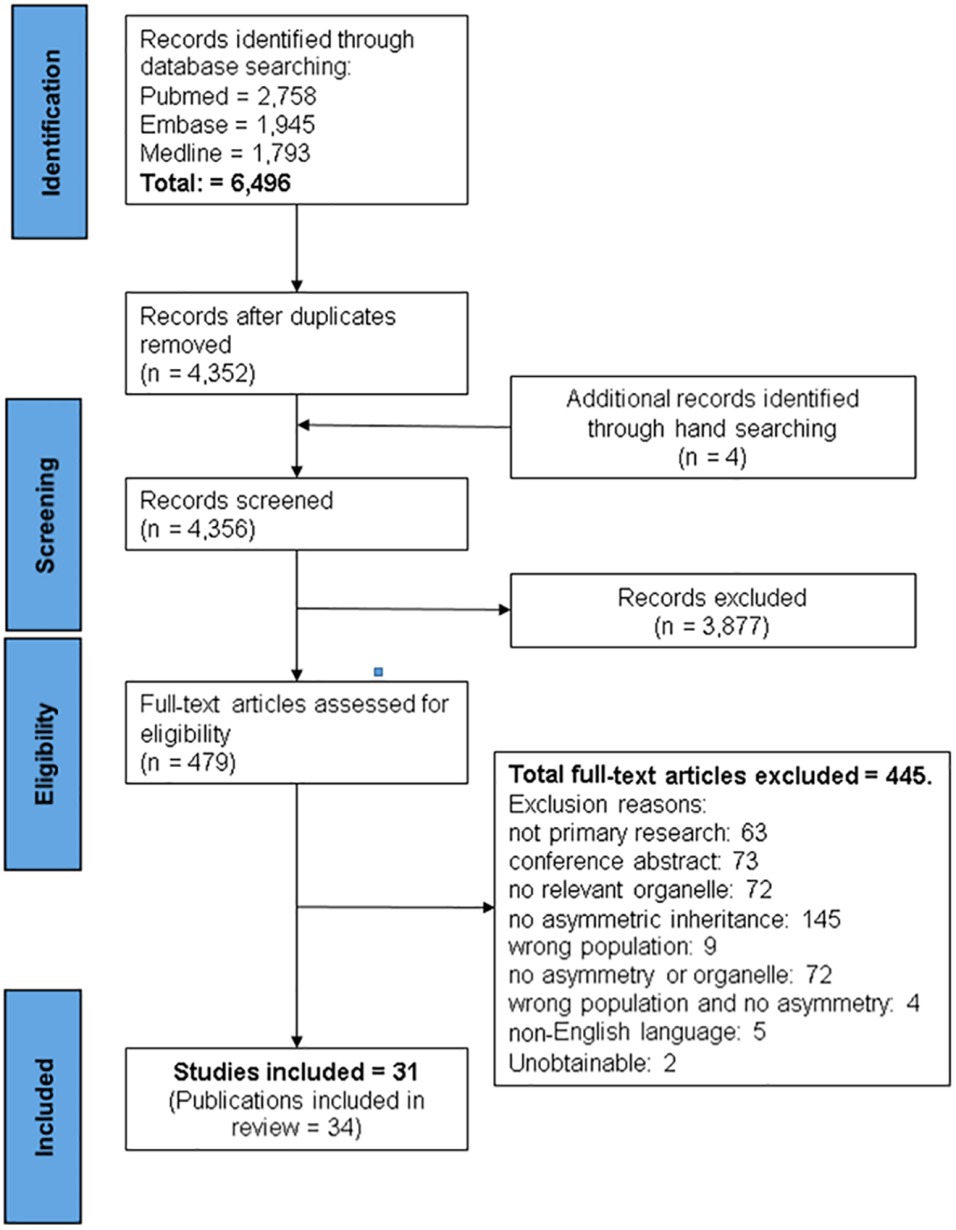
PRISMA flow diagram of the study selection process.

A summary of the identification and selection of studies for inclusion in this review is presented in Fig 1, in accordance with the PRISMA statement [15]. Literature searches of electronic databases retrieved 6,496 articles and hand searching identified four additional articles. After de-duplication 4,356 titles/abstracts were screened and 3,877 papers were excluded as having no relevance to the review. Full papers of 479 potentially relevant references were selected for further examination. Of these, 445 papers were excluded after reading the full paper, the reasons for exclusion are provided in Fig 1. Thirty one studies (34 publications) met the inclusion criteria.

### Asymmetric inheritance of organelles

Using systematic review methodology, we identified 31 studies describing the asymmetric inheritance of organelles after mitotic or meiotic division, in animal eukaryotic cells. Nine studies reported asymmetric inheritance for centrosomes, two for cilia, two for endoplasmic reticulum, six for endosomes, three for the midbody, three for mitochondria, four for P granules, two for proteosomes and two for spectrosomes (Table 1). Nine different organisms were identified for the model systems. Four studies used human models [20–23], the majority used *Drosophila* and mouse. Most studies used embryonic cells but three were in adult stem cells and five were in adult non-stem cells. Two studies of meiotic division were in oocytes; the remaining studies were of mitotic division. Three studies had two publications each [24–26], three studies had data for more than one organelle [22,27,28] and one study [23] had models for more than one organism. Publication dates ranged from 1991 to 2015. Only six (19%) studies reported that the result was repeated in another model [22,23,29–32]. The aim of the current review was to focus on the imprecision and quality of the included studies rather than presentation of the results in further detail.

**Table 1:** Characteristics and validity of studies showing asymmetric inheritance of organelles.

Organelle	Organism	Study ID	Meiosis or mitosis	Stem cell or non-stem cell	Marker	n	Model validity	Imprecision	Marker validity
**Centrosome **	Mouse	Wang 2009 [33]	mitosis	embryonic	Centrin 1	7	Low	High	Unclear
	Starfish	Tamura 2001 [34]	meiosis	oocyte	Phase mic.	58	Low	Unclear	Unclear
	Sea Urchin	Holy 1991 [35]	mitosis	embryonic	Anti-4D2	24	Low	Unclear	Unclear
	Drosophila	Conduit 2010 [36]	mitosis	embryonic	PACT, centrosomin	30	Low	Unclear	Unclear
		Januschke 2011, Rebollo 2007 [24,37]	mitosis	embryonic	PACT, centrobin/ASL	20	Unclear	Unclear	Unclear
		Yamashita 2007 [38]	mitosis	embryonic	PACT	331	Low	Unclear	Unclear
		Rusan 2007 [39]	mitosis	embryonic	Centrosomin	NR	Low	Unclear	Unclear
		Salzmann 2013 [27]	mitosis	embryonic	Centrobin	54	Low	High	Unclear
	Tubifex	Shimizu 1996 [40]	mitosis	embryonic	ɣ tubulin	NR	Low	Unclear	Unclear
**Centrosome (cilia) **	Mouse	Anderson 2009 [29]	mitosis	embryonic	Centrin, α tubulin	300	Unclear *	Unclear	Unclear
		Piotrowska-Nitsche 2012 [41]	mitosis	embryonic and adult non-stem	SStr3	30	Unclear	Unclear	Unclear
**Endoplasmic Reticulum **	Mouse	Dalton 2013 [28]	meiosis	oocyte	Dil18	12	Low	Unclear	Unclear
	Drosophila	Smyth 2015 [42]	mitosis	embryonic	Sec 61α	16	Low	Unclear	Unclear
**Endosome **	Human	Beckmann 2007 [20]	mitosis	adult stem cell	CD53, CD63, CD71	131	Low	Unclear	Unclear
	Drosophila	Coumailleau 2009 [43]	mitosis	embryonic	SARA	18	Low	Unclear	Unclear
		Emery 2005 [44]	mitosis	embryonic	Rab 11	50	Unclear	Unclear	Unclear
		Kressman 2015 [45]	mitosis	embryonic	SARA	27	Unclear	Unclear	High
		Loubery 2014 [46]	mitosis	embryonic	SARA	24	Low	Low	Unclear
		Montagne 2014 [47]	mitosis	adult stem cell	SARA	28	High	Unclear	Unclear
**Midbody **	Human	Kuo 2014 [30]	mitosis	embryonic and adult non-stem	MKLP1	23	Unclear *	Unclear	Unclear
	Monkey	Goss 2008 [31]	mitosis	adult non-stem	MKLP1	375	Unclear *	Unclear	Unclear
	Drosophila	Salzmann 2013 [27]	mitosis	embryonic	Pavarotti	200	Low	Unclear	Unclear
**Mitochondria**	Human	Katajisto 2015 [22]	mitosis	adult stem cell	Oomp25	5	High *	High	Unclear
	Mouse	Rivolta 2002 [48]	mitosis	embryonic	Mab48	921	Unclear *	Low	Unclear
	Mouse	Dalton 2013 [28]	meiosis	oocyte	Mito-GFP	15	Low	Unclear	Unclear
**P granule **	C. Elegans	Gallo 2010 [49]	mitosis	embryonic	PGL1	3	Low	High	Low
		Rose 1998, Basham 1999 [25,50]	mitosis	embryonic	OICID4, K76	NR	Low	Unclear	Unclear
		Boyd 1996 [51]	mitosis	embryonic	OICID4	42	Low	Unclear	Unclear
		Pang 2004 [52]	mitosis	embryonic	K76	NR	Low	Unclear	Unclear
**Proteosome **	Human	Ogrodnik 2014 [23]	mitosis	adult non-stem cell	von Hippen-Lindau	42	Unclear *	Unclear	Unclear
	Mouse	Chang 2011 [32]	mitosis	adult non-stem cell	Proteoosome 20s α1	125	Low *	Unclear	Unclear
	Hamster	Ogrodnik 2014 [23]	mitosis	embryonic	von Hippen-Lindau	42	Unclear*	Unclear	Unclear
**Spectrosome/ fusome **	Drosophila	de Cuevas 1998 [53]	mitosis	embryonic	hts	NR	Unclear	Unclear	Unclear
		Lin 1995, Deng 1997 [26,54]	mitosis	embryonic	α spectrin	10	Low	High	Unclear

If there were multiple results we have listed the result with the most technical repeats.

* The result was reproduced in more than one model; primary model reported here, see S4 Table for details.

Mic = microscopy; n = technical repeats.

### Validity

All studies were assessed for risk of bias using the SYRCLE checklist [3], this is the only risk of bias tool for non-clinical studies. Using SYRCLE we judged most domains to be unclear or not reported (S5 Table). SYRCLE is based on the risk of bias tools created for randomised controlled clinical trials [14], we found that this was not applicable to the design of basic scientific studies. Although the signalling questions of SYRCLE are not irrelevant to basic science studies they do not use a language that is meaningful to laboratory scientists nor do they critique all issues relevant to the biases of basic research. SYRCLE is relevant for preclinical mouse studies which are designed on the same basis as clinical trials. Laboratory based research does not formally randomise experiments, nor use allocation concealment or blinding. Good practice should ensure whoever sets up the experiment does not measure the outcome. In addition, all efforts are made to ensure that the experiment and controls are treated alike during all stages of the experiment. Based on our experience of designing and implementing laboratory experiments, we created three new tools to judge the quality of the research question under review. We believe that the fundamental biases in basic research derive from the choice of model (and its functional validation if necessary) and from demonstrating the experimental outcome is valid for the effect you want to measure. The reliability of a result is as important as the quality, therefore we also interrogated the statistical reporting. Therefore, we created tools to judge the choice and validation of the model system(s) and the organelle marker(s), and in addition, the reliability or imprecision of the reported result. The summary judgements are provided in Table 1 and the tools and detailed judgements are given in S2–S4 Tables.

### Imprecision

An imprecision tool was created, to judge how well the authors reported statistical variability, sample size and statistical methodology (S2 Table). We determined the minimum requirement for low risk was that the authors reported technical repeats, interassay repeats and variability. Only two studies (7%) had an overall judgement of low risk [46,48], the majority of studies (77%; 24/31) were 'unclear'. Five (16%) studies had an overall rating of 'high concern'; the main reasons for which were low technical repeats (<10).

**Fig 2:**
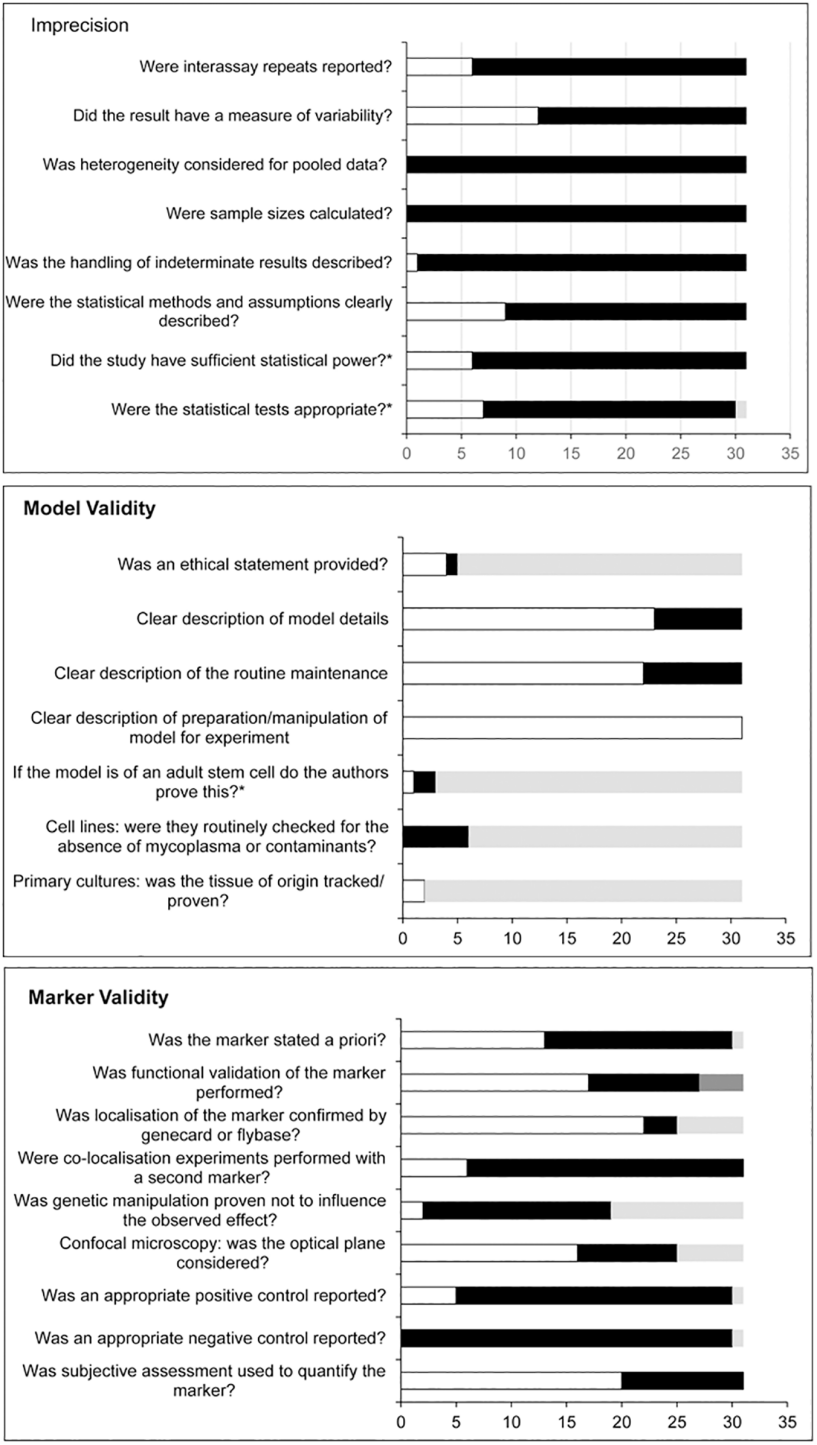
Assessments of imprecision, model validity and marker validity. Black bars = number of studies for judgements of 'no' or 'not reported'. White bars = number of studies for judgements of 'yes'. Light grey bars = number of studies 'not applicable' for question (unclear for imprecision). Dark grey bars = number of studies where the question was justified with a reference. *based on our judgements, see relevant tool for explanation. Judgements were based on the author's reported organelle marker, primary model and the result with the highest technical repeat.

Analysis of the individual signalling questions indicated that 84% of studies reported a technical repeat (Table 1), 19% of studies reported interassay repeats and 39% reported a measure of variability. None of the studies considered whether the pooling of data from interassay repeats was appropriate (heterogeneity) and none of the studies calculated a sample size. Only one study described how indeterminate results were handled and only 29% clearly described the statistical methods (summarised in Fig 2, details in S2 Table).

The lack of sample size calculations, technical repeats, interassay repeats and variability measures is of great concern. These are the basic determinants for demonstrating the reliability of a result and are essential for clinical translation or for deriving firm conclusions. For the purposes of a working tool we decided that an appropriate technical repeat should be greater than 10 and that there should be at least three interassay repeats. This number was determined by Charan and Kantharia 2013 [55], who suggested that in the absence of a power calculation a law of diminishing returns can be applied for a crude estimation of sample size in animal studies. We would like to emphasise that this is not a replacement for sample size calculations and authors should strive to perform pilot studies to identify the best sample size for their study. We determined that only 19% of studies had a sufficient sample size (Fig 2). We also determined whether the statistical methods employed were appropriate or not. For this review the judgement was based on whether the authors used appropriate t- tests (paired or unpaired). Although 29% of studies reported statistical methods, only 23% of studies were judged to have an appropriate statistical test (Fig 2). For two of the nine studies, which reported statistical methods [23,36] it was unclear whether the result was derived from pooling interassay repeats or not and therefore we could not judge if the test was appropriate. It should be noted that other tests besides the t–test could be appropriate for analysis but these were not reported by the included studies and therefore the guidance here concentrated on the t-test.

### Model validity

A model validity tool was created (S3 Table), to judge how well the authors reported the details of the model and whether the model was valid for the research question (e.g. if a stem cell was investigated, did the authors prove stem cell function?). A variety of two and three dimensional models using whole organisms, primary cultures, tissue and cell lines were employed in the studies. We determined the minimum requirement for low risk was that all domains were clearly reported and that there were no additional concerns. Most studies (61%; 19/31) were judged to be valid, ten studies (32%) were judged to be unclear for model validity. Two studies (7%) were judged to have high concern for model validity [22,47]; both models were described as 'stem cell-like' without providing experimental evidence to confirm this (e.g. pluripotency and self-renewal).

Analysis of the individual signalling questions (Fig 2, details in S3 Table) indicated that 83% (5/6) of relevant studies reported an ethical statement (whole mouse models or human tissue). 74% reported clear descriptions of the model, 71% described routine maintenance and 100% provided clear details of how the model was manipulated for experimentation. Such reporting details are important to allow a comparison of results between similar models and to clearly understand how the experiments were performed and whether sufficient details were available to repeat the experiment. Closer validation of the studies indicated that only one of three stem cell models provided evidence of stem cell function. None of the six studies which employed cell lines reported that they routinely checked for the absence of contaminants in the cultures. This is an important consideration because cell lines can be many years old and may have deviated from the original clone or become contaminated by other cells or microorganisms if not strictly maintained. Both studies using primary cultures provided data to confirm the tissue origin of the cells.

With regards to the model, other points to consider when establishing which studies provide the most valid data include: the cellular complexity of the model and the proximity of the phylogenic order to the organism stated in the research question. The best models are those of whole organisms or tissue, where the physiology, architecture and cellular niches are present and recapitulate tissue. In contrast, cell cultures grown in two dimensions do not recapitulate tissue architecture. This can be improved with the use of three dimensional cultures or multiple cell types grown in a co-culture system. Therefore, the best human evidence for the 'asymmetric inheritance of organelles’ in human models was derived from endosomes (in adult haematopoietic stem cells), the mid body (embryonic stem cells, adult epithelial cell lines) and proteosomes (adult embryonic cell line) [20,23,30]. This evidence largely came from two-dimensional cell culture models which illustrates that, for clinical translation, there is often a trade-off between how direct the evidence is and the complexity of the model. Before basic research is translated to the clinic there should always be evidence in the most valid human model available (cultures or tissue).

### Marker validity

A marker validation tool was created to judge the ability of the selected marker to correctly identify the organelle (S4 Table). This tool was also used to evaluate whether a marker can correctly identify a given cell (if relevant). We determined the minimum requirement for low risk was that all domains were clearly reported and there were no additional concerns.

One study was judged to have clear validity [49], the majority of studies were judged to be unclear (96.5%; 30/31), indicating that for most studies there were insufficient details to judge if the markers were appropriate. One study (3.5%) was judged to have high concern for marker validity [45]; in this study the asymmetry of SARA-marked early endosomes was reported, however the localisation of SARA was validated using co-localisation with Rab5c, but Rab5c did not exhibit asymmetry and therefore a conflict exists between these results.

Analysis of the individual signalling questions (Fig 2, details in S4 Table) indicated that only 43% of studies reported the choice of marker ‘a priori’. In basic research this is not unusual, authors tend not to state whether the marker used in a study was the only marker employed or whether several markers were tried and only one worked (and was reported). In a clinical review this would be referred to as 'selective outcome reporting’. This is still an important consideration and bias for basic research. Authors should be encouraged to report negative results for additional markers, as this has relevance for the validity or reliability of the measured result. 55% of studies reported a functional validation for the marker. Markers for the centrosome, mid body and spectrosome were rated as valid because their cellular location (using microscopy) confirmed their function. Mitochondrial and proteosomal markers were generally confirmed by co-localisation with a second marker or by functional validation. However, the absence of marker validation for many studies led the review authors to obtain further evidence from GeneCards and Flybase databases which confirmed the function of 88% of relevant markers. Only six studies used colocalization with a second marker to confirm the cellular location.

In studies which used genetic manipulation to mark the organelle only two studies (6%) provided data to confirm that the manipulation did not influence the result [29,44]. Most studies reported clear methods for imaging techniques, but 9 of 25 (36%) confocal studies did not consider the focal plane.

Very few studies (17%) reported positive controls and none reported negative controls. The reporting of controls should be fundamental, but their reporting is often overlooked often due to the space restriction applied by scientific journals, compounded by the complexity and number of experiments reported therein. We believe that authors should still report these results in appendices, especially when imaging techniques are used or threshold values are applied to decide between a negative or positive result. The importance of threshold values is illustrated by the different methods used to quantitate asymmetry (of the marker). Asymmetric inheritance was quantified by subjective assessment in 65% (20/31) of included studies (Fig 2; S4 Table); whereby the authors used visual inspection of the marker to decide whether asymmetry had occurred. Subjective assessment is not considered reliable unless verified by an independent researcher, but verification was not reported by any study. Table 2 summarises the studies which used quantitative methods to measure asymmetry; based on the pixel intensity of fluorescent microscopic images.

**Table 2:** Methods to quantify asymmetry.

Author reports asymmetry			Author reports no asymmetry			
Marker	Quantitative Threshold Based On:		Marker	Quantitative Threshold Based On:		
	Ratio of pixels between daughter cells	% of cells displaying asymmetry		Ratio of pixels between daughter cells	% of cells displaying asymmetry	
Anti-4D2	> 1	No	NA			[35]
Dil18	1.92 **	No	NA			[28]
Sec 61α	1.2 **	No	NA			[42]
SARA	15	No	PtdIns(3)P, Rab5	3	No	[43]
SARA	1.5	68%	Rab5c, Rab 7, Rab 11	1.5	7–9%	[45]
SARA	4.8^1^	No	NA			[46]
SARA	3^2^	No	Rab 7, Rab 11	1.9	No	[47]
Oomp25	5.6	No	Oomp25	1.25	No	[22]
Mab48	> 90% of one of two cell regions is devoid of labelling*	0.8–11.5%	Mab48	> 90% of one of two cell regions is devoid of labelling*	0.1–0.33%	[48]
Mito GFP	3.5 **	No	NA			[28]
PGL1	1.5	No	NA			[49]
Proteoosome 20s	1.5	No				[32]

* Assumes cell is dividing.

** fold enrichment either side of spindle.

^1^ Ratio based on reporting of 83% of fluorescence in mother cell goes to one daughter.

^2^ Ratio based on reporting of 75% of fluorescence in mother cell goes to one daughter.

Thresholds were not predefined, but were those reported y the author. If multiple thresholds were reported we selected the lowest (or range).

All the studies using quantitative methods used a threshold to determine whether asymmetry had occurred. The threshold was based on the ratio between pixels measured in one daughter cell compared to the other. A deviation away from 1 (or 1:1) was sufficient for one study to indicate asymmetry, but the fold increase reported ranged from 1 to 15 overall. The thresholds were not predefined and illustrate that, what one authors considers as asymmetry may not be sufficient for another researcher. For example, if we select a ratio of 3 as the threshold only 5/11 studies in Table 2 would meet this criterion. Two of the studies based a positive result of the proportion of cells in a given population which displayed asymmetric distribution of the marker between two daughter cells. One study [45] reported that asymmetry had not occurred because it was only observed in 7–9% of the cell population, whilst in another study this result would have been considered as asymmetry (0.8 to 11.5% of the population)[48]. Therefore, systematic reviewers need to carefully interrogate how the outcome is defined and should investigate closely the potential heterogeneity in the modes of measurement and any thresholds applied. Consideration of the outcome definition is a consideration for the reviewer but is not a limitation of the study; how well the results can be combined is influenced by the heterogeneity inherent in these descriptions.

### Risk of bias assessment and validity in other cell based systematic reviews

**Fig 3:**
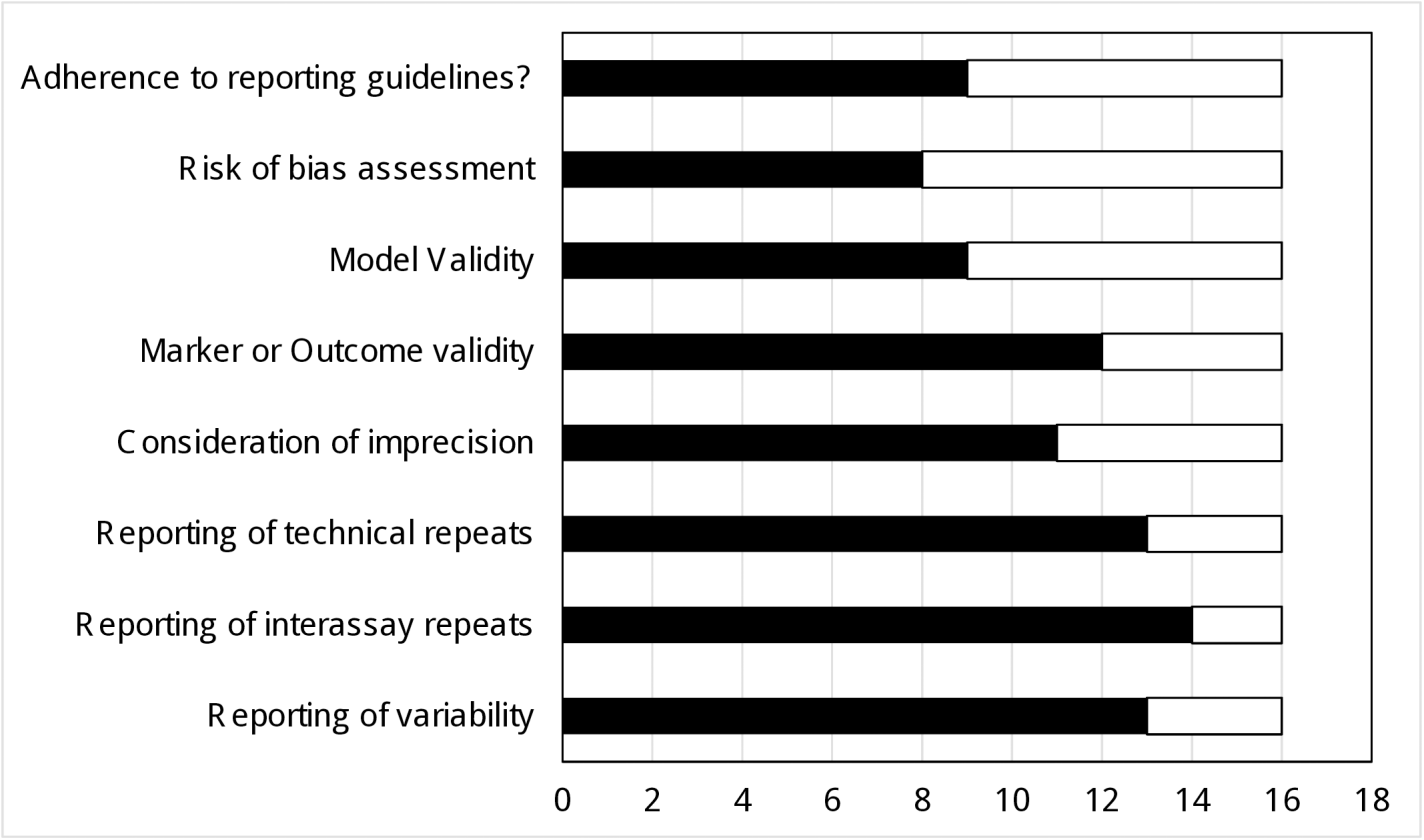
Assessments of guideline adherence, risk of bias, imprecision and validity in similar systematic reviews. Black bars = number of reviews not providing information for validity reporting/ assessment. White bars = number of reviews which did provide information for validity reporting/ assessment.

To establish how well other systematic reviews of based research, including cell assays, have assessed the quality of the included studies we performed a second review. We identified 16 systematic reviews (flow diagram in S1 File). We aimed to identify the use of formal or informal reporting guidelines, risk of bias tools or consideration of marker and model validity. We gave special focus to imprecision. The results are summarised in Fig 3, and further details are presented in S6 Table.

Fifty six percent of the reviews reported adherence to PRISMA or ARRIVE reporting guidelines. Only 44% reported a formal risk of bias assessment (Cochrane, GRADE, SYRCLE, Tool for Studies with Diverse Designs), one reported their own grading system [56]. Of the four reviews reporting Cochrane or SYRCLE tools it was notable that the majority of domains were rated as unclear, reiterating that the study designs are not based on randomisation, allocation concealment or blinding [57–60]. This finding agrees with our own assessments and indicates that basic research requires its own risk of bias tools. 56% of the reviews did not consider how relevant or valid the models were to the research question. Five reviews discussed the limitations of cell lines or culture methods [61–65], one review considered whether the origin of the primary culture was tracked [66]. 75% reviews did not consider how valid the markers were. One review did consider the marker validity [66], three discussed outcome validity or quantification methods [57,62,63]. 69% of reviews did not consider the impact of imprecision; those that did consider any aspect of imprecision reported minimal details. If we consider the minimum requirements for our imprecision tool, only 19% of reviews reported technical repeats, 13% reported interassay repeats and 19% reported the statistical variability of the result. These results clearly demonstrate that current systematic reviews carried out in basic research are not assessing the quality of the research nor the imprecision of the results. We found that the reviews which do assess quality are not conforming to any useful standards. Encouragingly some of the signalling questions we have devised for our model and marker validity tools where identified as important considerations in other reviews.

## Conclusions

Using a systematic review of basic science studies, we have identified the asymmetric inheritance of several organelles occurs across many eukaryotic animal species. However, we also found that current risk of bias tools are not adequate and are not widely used in basic science systematic reviews. Improvements are needed in the reporting of basic science and to improve the tools to judge the quality of basic scientific studies.

To overcome issues surrounding quality we have developed new tools to assess the imprecision of outcomes, marker validity and model validity. In the review presented here, these tools demonstrate that authors do not present sufficient information to judge the statistical reliability of their results. Most models were valid, except when specific cell types were employed (e.g. adult stem cells). Half of the included studies did not validate the function of their marker and only a small minority reported controls. These tools represent a first step towards the improvement of basic science appraisals using systematic review. We believe that the tools created here will be broadly applicable to many other fields and we encourage other researchers to use them as a starter for other assays e.g. cell division or microarray experiments.

Our results indicate that the requirements for publishing basic research are not rigorous, especially with regards to the statistical reliability of the results. The studies in this review were published between 1991 to 2015 in a range of journals. Although attempts are being made to improve basic science reporting (see checklists for Nature) this guidance is going unnoticed or is not a strict requirement for publication. As ‘sloppy science’ becomes increasingly recognised [67–69] it is likely that scientists will give more credence to the evaluation and reporting of their work.

This systematic review also highlights the need for basic science reviewers to identify the best quality evidence and the most reliable, rather than just a summary of results. Our evaluation of other basic science systematic reviews indicated that none successfully considered the statistical reliability of the results and few judged the quality. We firmly believe that the ‘best’ results are dependent on both the quality of the research and the statistical reliability. In addition, the reviewer must closely interrogate the outcome definitions, to determine whether they can be combined or compared across studies. The best systematic reviews are not just a process but indicate the best available evidence for a given question. This is especially important for research that will be used for clinical translation. A systematic review evaluating statistical reliability and validity will provide better evidence to ensure greater clinical success, reduce early investment costs and reduce unnecessary animal experimentation.

## Supporting information

S1 File PRISMA checklist. (DOC)

S2 File Search strategies. (DOCX)

S1 Table Inclusion and exclusion criteria for screening. (DOCX)

S2 Table Imprecision tool and assessments. (DOCX)

S3 Table Model validity tool and assessments. (DOCX)

S4 Table Marker validity tool and assessments. (DOCX)

S5 Table Risk of bias (SYRCLE). (DOCX)

S6 Table Validity assessments and adherence to reporting guidelines in systematic reviews of cellular research. (DOC)
