# Supplementary material for: A systematic review of the asymmetric inheritance of cellular organelles in eukaryotes: A critique of basic science validity and imprecision
Source: PLoS One. 2017 May 31;12(5):e0178645. doi: 10.1371/journal.pone.0178645 (PMC5451095; doi:10.1371/journal.pone.0178645)
Supplement: S2 File — (DOCX) [file pone.0178645.s002.docx]

# S2 File:

# A: Search Strategies for Asymmetric inheritance

**Embase**

1 asymmetric cell division/ (534)

2 (unequal or polari$ or non-random or asymmetric).ti,ab. (125415)

3 1 or 2 (125794)

4 exp cell division/ (173168)

5 (inheritance or segregate$ or partition$ or mitosis or apportion$ or division$ or divide$).ti,ab. (670613)

6 4 or 5 (788569)

7 exp cell organelle/ (413109)

8 (organelle$ or nucleus or golgi or endoplasmic reticulum or mitochondria or proteasome$ or lysosome$ or centrosome$ or centriole$ or autophagosome$ or vacuole$ or sarcoplastic reticulum or exosome or microtubule organi?ing cent? or spindle pole body or mitochondrion or peroxisome$).ti,ab. (534678)

9 7 or 8 (762228)

10 3 and 6 and 9 (1945)

**Medline**

1 Asymmetric Cell Division/ (182)

2 (unequal or polari$ or non-random or asymmetric).ti,ab. (140590)

3 1 or 2 (140620)

4 exp cell division/ (236854)

5 (inheritance or segregate$ or partition$ or mitosis or apportion$ or division$ or divide$).ti,ab. (458379)

6 4 or 5 (656695)

7 exp Organelles/ (432444)

8 (organelle$ or nucleus or golgi or endoplasmic reticulum or mitochondria or proteasome$ or lysosome$ or centrosome$ or centriole$ or autophagosome$ or vacuole$ or sarcoplastic reticulum or exosome or microtubule organi?ing cent? or spindle pole body or mitochondrion or peroxisome$).ti,ab. (444918)

9 7 or 8 (712902)

10 3 and 6 and 9 (1704)

**Medline In-Process & Other Non-Indexed Citations**

**Medline Daily Update**

1 Asymmetric Cell Division/ (1)

2 (unequal or polari$ or non-random or asymmetric).ti,ab. (52832)

3 1 or 2 (52832)

4 exp cell division/ (45)

5 (inheritance or segregate$ or partition$ or mitosis or apportion$ or division$ or divide$).ti,ab. (52909)

6 4 or 5 (52930)

7 exp Organelles/ (219)

8 (organelle$ or nucleus or golgi or endoplasmic reticulum or mitochondria or proteasome$ or lysosome$ or centrosome$ or centriole$ or autophagosome$ or vacuole$ or sarcoplastic reticulum or exosome or microtubule organi?ing cent? or spindle pole body or mitochondrion or peroxisome$).ti,ab. (28193)

9 7 or 8 (28300)

10 3 and 6 and 9 (89)

**Pubmed**

| Search | Query | Items found |
| --- | --- | --- |
| #16 | Search #4 AND #9 AND #15 | 2758 |
| #15 | Search #12 or #14 | 825473 |
| #14 | Search Search organelle* or nucleus or golgi or endoplasmic reticulum or mitochondria or proteasome* or lysosome* or centrosome* or centriole* or autophagosome* or vacuole* or sarcoplastic reticulum or exosome or microtubule organising center or spindle pole body or mitochondrion or peroxisome*[tiab] | 739655 |
| #12 | Search "organelles"[Mesh] | 420092 |
| #9 | Search #5 or #8 | 1666034 |
| #8 | Search [inheritance or segregate* or partition* or mitosis or apportion* or division* or divide*[tiab] | 1652122 |
| #5 | Search "Cell Division"[Mesh] | 232322 |
| #4 | Search #2 or #3 | 575606 |
| #3 | Search unequal or polari* or non-random or asymmetric[tiab] | 575582 |
| #2 | Search "Asymmetric Cell Division"[Mesh] | 156 |

**B: SEARCH STRATEGIES FOR SYSTEMATIC REVIEWS OF BASIC CELLULAR RESEARCH**

**Embase, Ovid MEDLINE(R) Epub Ahead of Print, In-Process & Other Non-Indexed Citations, Ovid MEDLINE(R) Daily and Ovid MEDLINE(R)**

1 cell.ti. (1871770)

2 systematic review.ti. (145704)

3 1 and 2 (2266)

4 remove duplicates from 3 (1446)

5 (cell or cells).ti. and 2 (2620)

6 remove duplicates from 5 (1667)

1 (eukaryote or eukaryotes or eukaryotic).ti,ab. (199027)

2 systematic review.ti. (145704)

3 1 and 2 (8)

4 remove duplicates from 3 (5)

**Pubmed**

| Search | Query | Items found |
| --- | --- | --- |
| #11 | Search #10 OR #9 | 968 |
| #10 | Search #6 AND #5 | 964 |
| #9 | Search #8 AND #5 | 4 |
| #8 | Search #2 OR #3 OR #4 | 94589 |
| #6 | cell[Title] | 828343 |
| #5 | systematic review[Title] | 64777 |
| #4 | eukaryotic[Title/Abstract] | 69627 |
| #3 | eukaryotes[Title/Abstract] | 29191 |
| #2 | eukaryote[Title/Abstract] | 4043 |

**C: Flow diagram FOR SYSTEMATIC REVIEWS OF BASIC CELLULAR RESEARCH**

**Total full-text articles excluded = 9.** Exclusion reasons:

No cell based assays: 4

Clinical outcomes: 2

Duplicates: 2

Unobtainable: 1

**Publications included in review = 16**

Full-text articles assessed for eligibility
(n = 25)

Records excluded
(n = 2,600)

Records screened
(n = 2,625)

Records after duplicates removed
(n = 2,625)

## Identification

## Eligibility

## Included

## Screening

Records identified through database searching:

Pubmed: 968

Embase, Medline = 2628

**Total: 3,596**
