# Supplementary material for: A systematic review of the asymmetric inheritance of cellular organelles in eukaryotes: A critique of basic science validity and imprecision
Source: PLoS One. 2017 May 31;12(5):e0178645. doi: 10.1371/journal.pone.0178645 (PMC5451095; doi:10.1371/journal.pone.0178645)
Supplement: S1 Table — (DOCX) [file pone.0178645.s003.docx]

**S1 Table. Inclusion and exclusion criteria for screening (asymmetric inheritance of organelles)**

|  | Include | Exclude |
| --- | --- | --- |
| Population | Normal eukaryotic cells | Prokaryotes  Cancer/ disease  Plants, algae, fungi |
| Outcome | Asymmetric inheritance following meiosis or mitosis | Intracellular polar distribution of organelles.  Asymmetric localisation of organelles |
| Organelles | Nucleus  Golgi/ trans Golgi network  Endoplasmic reticulum  Sarcoplastic reticulum  Mitochondria  Vacuole  Proteosome  Lysosome  Centrosome/ microtubule organising centre/ centriole  Autophagosome  Exosome  Spindle pole body  Peroxisome  Fusome  Spectrosome | Specific proteins, genes, DNA  Transcription factors  Cell size  Chromosomes, chromatids (BrdU labelling) |
| Study design | Original research | Reviews  Conference abstracts  Foreign language articles |

**Inclusion and exclusion criteria for screening (systematic reviews of basic cellular research)**

|  | Include | Exclude |
| --- | --- | --- |
| Population | Eukaryotic cells |  |
| Outcomes | Cell based assays for any outcome | Diagnostic, prognostic, clinical outcomes. |
| Study design | Systematic review |  |
