# Supplementary material for: A systematic review of the asymmetric inheritance of cellular organelles in eukaryotes: A critique of basic science validity and imprecision
Source: PLoS One. 2017 May 31;12(5):e0178645. doi: 10.1371/journal.pone.0178645 (PMC5451095; doi:10.1371/journal.pone.0178645)
Supplement: S2 Table — (DOCX) [file pone.0178645.s004.docx]

**S2 Table. Imprecision tool**

|  | **Signalling question** | **Notes** | **Answer** |
| --- | --- | --- | --- |
| **TECHNICAL REPORTING** | **1. How many technical repeats were performed per experiment?** | Intra - assay variability | Free text |
|  | **2. Observer variability: Did the experiment give the same result when repeated?** | Inter-assay variability | Free text |
|  | **3. Is it clear whether the technical repeat is true or a combination of technical and observer variability?** |  | Yes/ no/ not applicable/ unclear or not reported |
|  | **4. Did the result include a measure of variability? Or was the data presented as a scatter plot?** | EB = error bars (unclear error), SE = standard error, SEM = standard error of the mean, SD = standard deviation  CI = confidence intervals | Free text |
|  | **5. Did the authors pool data between experiments? If so, was heterogeneity measured to test that pooling was appropriate?** | (Important when using multiple patient /animal samples) | Yes/ no/ not applicable/ unclear or not reported |
|  | **Overall reporting rating** | Low= no concern for bias. Unclear = insufficient data to make a judgement. High risk = there is a concern of high risk. If 1,2 and 4 are fulfilled this can be given a low rating for this review. | **Low/ Unclear/ High** |
| **SAMPLE SIZE** | **6. Were sample sizes calculated?** | For the given experiment/effect did the authors calculate the number of repeats that would be required for significance? | Yes/ no/ not applicable/ unclear or not reported |
|  | **7. How were indeterminate results, missing results, and outliers handled?** |  | Free text |
|  | **8. Did the study have sufficient statistical power?** | Yes: clearly meets the sample size.  Likely: >10 repeats with inter-assay repeats. Unclear: >10 repeats, no inter-assay repeats.  No: ≤10 technical repeats. | Yes/ no/ unclear / likely |
|  | **Did the study have sufficient statistical power? Justification** | Based on questions 6-8 | Free text |
|  | **Overall sample size rating** | Low= no concern (or likely statistical power or Unclear statistical power plus variability reported).  Unclear: not enough information to make judgement and no high risk for 6-8.  High risk: there is a concern of high risk for 6-8. | **Low/ Unclear/ High** |
| **STATISTICAL TEST** | **9. Description of statistical methods and assumptions.** | P S TT = Paired student t-test; US TT = unpaired student t test; x2-test =XT; Fishers exact test = FET; others posssible | Free text |
|  | **10. Were the statistical tests appropriate?** | In this review t- tests were the predominant test (other statistical tests are possible). A paired t test is the most appropriate test for comparisons between the same cell lines or non-human models, because these are assumed to be homogeneous populations. An unpaired t test should be used for comparisons between primary cultures, human tissues or different mutants or strains, because these will be heterogeneous populations. | Yes/ no/ not applicable/ unclear or not reported |
|  | **Were the statistical tests appropriate? Justification** |  | Free text |
|  | **11. Evidence of data dredging** | https://en.wikipedia.org/wiki/Data_dredging | Yes/ no/ not applicable/ unclear or not reported |
|  | **Statistical test rating** | **Low= no concern. Unclear = not enough information to make judgement. High risk = there is a concern of high risk** | **Low/ Unclear/ High** |
|  | **Other Concerns** |  | Free text |

| **OVERALL RATING** | **Based on the judgements for the 3 domains above and any other concerns.**  **Low= all domains clearly reported. Unclear = Any domains are unclear, but not high risk. High risk = there is a concern of high risk** | **Low/ Unclear/ High** |
| --- | --- | --- |
| **JUSTIFICATION** | Text to justify final rating and any deviation from tools instructions | Free text |

**Imprecision Tool Assessments**

| **First Author Surname and Year** | **Experiment** | **1.** | **2.** | **3.** | **4.** | **5.** | **reporting rating** | **6.** | **7.** | **8.** | **sample size rating** | **9.** | **10.** | **11.** | **statistical test rating** | **Overall rating** | **justification** | |
| --- | --- | --- | --- | --- | --- | --- | --- | --- | --- | --- | --- | --- | --- | --- | --- | --- | --- | --- |
| **Centrosome** | | | | | | | | | | | | | | | | | | |
| Conduit 2010 | F1 | 30 | NR | UNR | No | UNR | *UNR* | no | NR | U | *UNR* | NR | UNR | UNR | *UNR* | **UNR** | | No variability with use of whole organism. No sample size calculation. |
| Holy 1991 | T1.  Mi vs. Ma | 24 | NR | UNR | No | UNR | *UNR* | no | NR | U | *UNR* | NR | UNR | UNR | *UNR* | **UNR** | | Unclear reporting of methods, variability. No sample size calculation, low technical repeats. |
| Januschke 2011 | F2 | 20 | NR | UNR | No | UNR | *UNR* | no | NR | U | *UNR* | NR | UNR | UNR | *UNR* | **UNR** | | No variability or quantitative data. No sample size calculation, low technical repeats. |
|  | F3 | 16 | NR | UNR | No | UNR | *UNR* | no | NR | U | *UNR* | NR | UNR | UNR | *UNR* | **UNR** | |  |
| Shimizu 1996 | F3 | NR | NR | UNR | No | UNR | *UNR* | no | NR | U | *UNR* | NR | UNR | UNR | *UNR* | **UNR** | | Unclear reporting of methods, variability and no technical repeats reported. No sample size calculation |
| Tamura 2001 | F2 | 58 | NR | UNR | No | UNR | *UNR* | no | NR | U | *UNR* | NR | UNR | UNR | *UNR* | **UNR** | | Unclear reporting of all methods and variability. No sample size calculation but 58 repeats. |
|  | T1.  PB1 | 22 | NR | UNR | No | UNR | *UNR* | no | NR | U | *UNR* | NR | UNR | UNR | *UNR* | **UNR** | | Unclear reporting of methods, variability. No sample size calculation performed, low technical repeats. |
|  | T1.  PB2 | 50 | NR | UNR | No | UNR | *UNR* | no | NR | U | *UNR* | NR | UNR | UNR | *UNR* | **UNR** | | Unclear reporting of methods, variability. No sample size calculation but 50 repeats. |
| Wang 2009 | F3 | 7 | NR | UNR | No | UNR | *UNR* | no | NR | no | *High* | NR | UNR | UNR | *UNR* | **High** | | Low technical repeats with use of whole organism. No sample size calculation. |
|  | F4 | NR | NR | UNR | No | UNR | *UNR* | no | NR | U | *UNR* | NR | UNR | UNR | *UNR* | **UNR** | | Unclear reporting of methods, technical repeats and no sample size calculation. |
| Yamashita 2007 | F1 | >100 | >3 | UNR | No | UNR | *UNR* | no | NR | L | *Low* | NR | UNR | UNR | *UNR* | **UNR** | | Unclear reporting of methods and quantitative data (variability), no sample size calculation. |
|  | F2 | 294-331 | >3 | UNR | No | UNR | *UNR* | no | NR | L | *Low* | NR | UNR | UNR | *UNR* | **UNR** | |  |
| Rusan 2007 | F2 | NR | NR | UNR | No | UNR | *UNR* | no | NR | U | *UNR* | NR | UNR | UNR | *UNR* | **UNR** | | Unclear reporting of methods, variability, no technical repeats reported, no sample size calculation. |
| Saltzmann 2013 | F5 | 54 | NR | UNR | No | UNR | *UNR* | no | NR | U | *UNR* | NR | UNR | UNR | *UNR* | **High** | | Unclear inter assay repeats, unclear statistical methods. No sample size calculation. Statistics only presented for cells showing asymmetry, not overall. |
| **Centrosome (cilia)** | | | | | | | | | | | | | | | | | | |
| Anderson 2009 | F2 | 35 | NR | UNR | No | UNR | *UNR* | no | NR | U | *UNR* | NR | UNR | UNR | *UNR* | **UNR** | | Unclear inter assay variability and statistical methods |
|  | F1B | 300 | 3 | yes | SEM | UNR | *Low* | no | NR | L | *Low* | NR | UNR | UNR | *UNR* | **UNR** | | Unclear statistical tests |
| Piotrowska-Nitsche 2012 | F2 | 27 | NR | UNR | No | UNR | *UNR* | no | NR | U | *Low* | P TT | yes | UNR | *Low* | **UNR** | | Unclear inter assay repeats and unclear variability. No sample size calculation. |
|  | F6 | 30 | NR | UNR | No | UNR | *UNR* | no | NR | U | *UNR* | P TT | yes | UNR | *Low* | **UNR** | | Possibility that only 1 embryo was used. No sample size performed calculation, low technical repeats. |
| **Endoplasmic Reticulum** | | | | | | | | | | | | | | | | | | |
| Dalton 2013 | F4 | 12 | NR | UNR | SEM | UNR | *UNR* | no | NR | U | *UNR* | P TT | UNR | UNR | *UNR* | **UNR** | | Low technical repeats with use of primary cultures. No sample size calculation. Unpaired t-test should be used if samples were from different mice, this is not reported therefore it is unclear if a paired test was appropriate. |
| Smyth 2015 | Fig 1b | 16 | NR | UNR | SEM | UNR | *UNR* | no | NR | U | *L* | TT | yes | UNR | *Low* | **UNR** | | No sample size calculations, low technical repeats, unclear inter assay repeats. T- test appropriate for identical cell populations. |
| **Endosome** | | | | | | | | | | | | | | | | | | |
| Beckmann 2007 | T3 - CD53 | 97 | 3 | UNR | SD | UNR | *Low* | no | NR | L | *Low* | P TT | UNR | UNR | *UNR* | **UNR** | | Unclear if result is based on one cord blood sample or more and whether it was appropriate to pool samples. No sample size calculation. |
|  | T3 - CD63 | 146 | 3 | UNR | SD | UNR | *Low* | no | NR | L | *Low* | P TT | UNR | UNR | *UNR* | **UNR** | |  |
|  | T3 - CD71 | 131 | 3 | UNR | SD | UNR | *Low* | no | NR | L | *Low* | P TT | UNR | UNR | *UNR* | **UNR** | |  |
| Coumailleau 2009 | F2c SARA | 18 | NR | UNR | SD | UNR | *UNR* | no | NR | U | *UNR* | NR | UNR | UNR | *UNR* | **UNR** | | Unclear method reporting and statistical tests. No sample size performed, low technical repeats. |
|  | F2c PtdIns  (3)P | 18 | NR | UNR | SD | UNR | *UNR* | no | NR | U | *UNR* | NR | UNR | UNR | *UNR* | **UNR** | |  |
|  | F2c Rab5 | 18 | NR | UNR | SD | UNR | *UNR* | no | NR | U | *UNR* | NR | UNR | UNR | *UNR* | **UNR** | |  |
| Emery 2005 | F1a Rab5 | NR | NR | UNR | No | UNR | *UNR* | no | NR | U | *UNR* | NR | UNR | UNR | *UNR* | **UNR** | | All domains were rated unclear/ not reported |
|  | F1a Rab7 | NR | NR | UNR | No | UNR | *UNR* | no | NR | U | *UNR* | NR | UNR | UNR | *UNR* | **UNR** | |  |
|  | F1,2 Rab11 | 50 | NR | no | No | UNR | *UNR* | no | NR | U | *UNR* | NR | UNR | UNR | *UNR* | **UNR** | | Unclear inter-assay repeats, no variability, no reporting of statistical methods |
| Kressmann 2015 | SF2g SARA | 27 | NR | UNR | No | UNR | *UNR* | no | ** | U | *UNR* | FET | yes | UNR | *Low* | **UNR** | | No sample size performed, low technical repeats, other experiments included inter assay repeats, but not reported for this experiment.  **outliers were excluded |
|  | SF2g Rab5c | 15 | NR | UNR | No | UNR | *UNR* | no | ** | U | *UNR* | FET | yes | UNR | *Low* | **UNR** | | Unclear if 15 technical repeats or 15 embryos, inter assay results were not reported. Unclear, no sample size performed. **outliers were excluded |
|  | SF2g Rab11a | 21 | NR | UNR | No | UNR | *UNR* | no | ** | U | *UNR* | FET | yes | UNR | *Low* | **UNR** | | Unclear inter assay repeats. No sample size calculation performed, low technical repeats. **outliers were excluded |
|  | SF2g Rab7 | 30 | NR | UNR | No | UNR | *UNR* | no | ** | U | *UNR* | FET | yes | UNR | *Low* | **UNR** | |  |
| Loubery 2014 | SF1b. | 24 | 4 | UNR | SEM | UNR | *Low* | no | NR | L | *Low* | TT | yes | UNR | *Low* | **Low** | | Based on 4 animals with 24 repeats in drosophila is likely to be acceptable in comparison to higher organisms. No sample size calculation. |
|  | F4d | 19 | 2 | UNR | SEM | UNR | *High* | no | NR | L | *Low* | TT | yes | UNR | *Low* | **High** | | Based on 2 animals only and unclear whether result is pooled data from both or just one. No sample size calculation. |
| Montagne 2014 | F1  SARA | 28 | NR | UNR | SEM | UNR | *UNR* | no | NR | U | *Low* | NR | UNR | UNR | *UNR* | **UNR** | | Inter-assay repeats not reported, statistical methods unclear, no sample size performed, low technical repeats. |
|  | F2  SARA | 4 | NR | UNR | No | UNR | *UNR* | no | NR | no | *High* | NR | UNR | UNR | *UNR* | **High** | | Based on 4 cells. No variability and no sample size calculation. |
|  | FS1c Rab7 | 6 | NR | UNR | No | UNR | *UNR* | no | NR | no | *High* | NR | UNR | UNR | *UNR* | **High** | | Based on 6 cells. No variability and no sample size calculation. |
|  | FS1b Rab11 | 3 | NR | UNR | No | UNR | *UNR* | no | NR | no | *High* | NR | UNR | UNR | *UNR* | **High** | | Based on 3 cells. No variability and no sample size calculation. |
| **Golgi** | | | | | | | | | | | | | | | | | | |
| Katajisto 2015 | F1B | NR | NR | UNR | EB | UNR | *UNR* | no | NR | U | *UNR* | TT | yes | UNR | *Low* | **UNR** | | Unclear sample size and calculation and variability. Paired or unpaired test would be appropriate for cell line |
| **Lysosome** | | | | | | | | | | | | | | | | | | |
| Katajisto 2015 | F1B | NR | NR | UNR | EB | UNR | *UNR* | no | NR | U | *UNR* | TT | yes | UNR | *Low* | **UNR** | | Unclear sample size or calculation and variability. Paired or unpaired test would be appropriate for cell line |
| Midbody | | | | | | | | | | | | | | | | | | |
| Goss 2008 | F5 | 375 | NR | UNR | SD | UNR | *UNR* | no | NR | U | *UNR* | NR | UNR | UNR | *UNR* | **UNR** | | Unclear inter assay repeats, unclear statistical methods |
| Kuo 2014 | F2d | 18 | > 3 | UNR | No | UNR | *UNR* | no | NR | L | *Low* | NR | UNR | UNR | *UNR* | **UNR** | | Unclear variability, low sample size, unclear statistical methods, no sample size calculation. |
|  | Text | 23 | > 3 | UNR | No | UNR | *UNR* | no | NR | L | *Low* | NR | UNR | UNR | *UNR* | **UNR** | | Unclear variability, unclear statistical methods, 23 repeats, no sample size calculation. |
| Saltzmann 2013 | F2 | >200 | NR | UNR | No | UNR | *UNR* | no | NR | U | *UNR* | NR | UNR | UNR | *UNR* | **UNR** | | Unclear inter assay repeats, unclear statistical methods |
|  | F3 | 61 | NR | UNR | No | UNR | *UNR* | no | NR | U | *UNR* | NR | UNR | UNR | *UNR* | **UNR** | |  |
| **Mitochondria** | | | | | | | | | | | | | | | | | | |
| Katajisto 2015 | F1B | NR | NR | UNR | EB | UNR | *UNR* | no | NR | U | *UNR* | TT | yes | UNR | *Low* | **UNR** | | Unclear sample size or calculation and variability. Paired or unpaired test would be appropriate for cell line |
|  | F3b | 5 | NR | UNR | EB | UNR | *UNR* | no | NR | no | *High* | TT | yes | UNR | *Low* | **High** | | Only 5 repeats. No sample size calculation. |
| Rivolta 2002 | F2 UB/OC1 | >1000 | 3 | UNR | SE | UNR | *Low* | no | NR | yes | *Low* | XT | yes | UNR | *Low* | **Low** | | t -test appropriate for cell line |
|  | F2 UB/UE1 | >1000 | 3 | UNR | SE | UNR | *Low* | no | NR | yes | *Low* | XT | yes | UNR | *Low* | **Low** | | t -test appropriate for cell line |
|  | F3f | NR | NR | UNR | No | UNR | *UNR* | no | NR | U | *UNR* | NR | UNR | UNR | *UNR* | **UNR** | | Unclear reporting of methods and sample size, no sample size calculation. |
| Dalton 2013 | F3 | 15 | NR | UNR | SEM | UNR | *UNR* | no | NR | U | *UNR* | P TT | UNR | UNR | *UNR* | **UNR** | | Low technical repeats with use of primary cultures, no sample size calculation. Unpaired t-test should be used if samples were from different mice, this is not reported therefore it is unclear if a paired test was appropriate. |
| **P granule** | | | | | | | | | | | | | | | | | | |
| Gallo 2010 | F2 | 3 | NR | UNR | SD | UNR | *UNR* | no | NR | no | *High* | NR | UNR | UNR | *UNR* | **High** | | No sample size calculation, only 3 embryos |
| Rose 1998 | F3. P1 | NR | NR | UNR | No | UNR | *UNR* | no | NR | U | *UNR* | NR | UNR | UNR | *UNR* | **UNR** | | No sample size, technical repeats or statistical methodology reported for wt |
|  | F3. P2 | NR | NR | UNR | No | UNR | *UNR* | no | NR | U | *UNR* | NR | UNR | UNR | *UNR* | **UNR** | |  |
| Boyd 1996 | F7 | 42 | NR | UNR | No | UNR | *UNR* | no | NR | U | *Low* | NR | UNR | UNR | *UNR* | **UNR** | | All domains were rated unclear for wt |
| Pang 2004 | F4a,b | NR | NR | UNR | No | UNR | *UNR* | no | NR | U | *UNR* | NR | UNR | UNR | *UNR* | **UNR** | | Lack of methodology reporting and technical repeats |
| **Spectrosome/ fusome** | | | | | | | | | | | | | | | | | | |
| de Cuevas 1998 | F3b | NR. | NR | UNR | No | UNR | *UNR* | no | NR | U | *UNR* | NR | UNR | UNR | *UNR* | **UNR** | | All domains were not reported. |
| Lin 1995 | F1,2 | 10 | NR | UNR | No | UNR | *UNR* | no | NR | no | *High* | NR | UNR | UNR | *UNR* | **High** | | Low technical repeats with use of a whole organism |
| **Proteosome** | | | | | | | | | | | | | | | | | | |
| Chang 2011 | F3a CD8+ | 74 | NR | no | No | UNR | *UNR* | no | NR | U | *UNR* | NR | NA | UNR | *UNR* | **UNR** | | Unclear inter-assay repeats, no variability, no reporting of statistical methods |
|  | F3b CD4+ | 125 | NR | no | No | UNR | *UNR* | no | NR | U | *UNR* | NR | NA | UNR | *UNR* | **UNR** | |  |
| Ogrodnik 2014 | F2a | 42 | NR | no | EB | UNR | *UNR* | no | NR | L | *Low* | UP TT | yes | UNR | *Low* | **UNR** | | Unclear inter-assay repeats |
| UNR= unclear or not reported; NR = not reported; U = unclear; L = likely; PS TT = Paired student t-test; US TT = unpaired student t test; x2-test =XT; Fishers exact test = FET; TT = t test or student’s t test. | | | | | | | | | | | | | | | | | | |
