# Supplementary material for: A systematic review of the asymmetric inheritance of cellular organelles in eukaryotes: A critique of basic science validity and imprecision
Source: PLoS One. 2017 May 31;12(5):e0178645. doi: 10.1371/journal.pone.0178645 (PMC5451095; doi:10.1371/journal.pone.0178645)
Supplement: S3 Table — (DOCX) [file pone.0178645.s005.docx]

**S3 Table. Model validity tool**

| **Signalling question** | **Notes** | **Answer** |
| --- | --- | --- |
| **1. Ethical statement** | Was an ethical statement provided for animal handling? | Yes/ NR – add details to justification |
| **2. Clear description of model details** | Brief description of basic model followed by: source, species, strain sex, developmental stage, age, passage number etc.). | Free text |
| **3. Is the model transgenic?** | Whether purchased or created. | Yes/no/unclear |
| **4. Clear description of the routine maintenance of the model** |  | Free text |
| **5. Further preparation of the model for experimentation** | Description of how model was manipulated to obtain result: to include preparation for imaging, how daughter or mother organelle were induced to differentiate. This should be used to make it clear how result was derived. | Free text |
| **6. If the model is of an adult stem cell do the authors prove this?** | Cells must be capable of dividing and renewing for long periods; undifferentiated; multipotent. | NA/ partial/ NR/ yes/ no – add details to justification. |
| **7. Cell lines: were they routinely checked for the absence of mycoplasma or contaminants?** |  | Yes/no/NR |
| **8. Primary cultures: was the tissue of origin tracked/ proven?** |  | Yes/no/NR |
| **9. Additional comments/ concerns** |  | NA/ partial/ No/ yes – add details to justification. |
| **Overall rating/reporting of model.** | **Low= all domains clearly reported, and there were no additional concerns. Unclear = Any domain was unclear, but not high risk. High risk = there is a concern of high risk. Note that for this review routine maintenance was not essential for low order organisms.** | **High/ Low/ Unclear or not reported** |
| **Justification** | **Text to justify why model was given unclear or high rating. Additional text for details regarding questions 1, 6-9.** | **Free text.** |

***Model Validity Tool Assessments***

| **Study ID** | **1.** | **2.** | **3.** | **4.** | **5.** | **6.** | **7.** | **8.** | **9.** | **Overall rating/**  **reporting of model** | **justification** |
| --- | --- | --- | --- | --- | --- | --- | --- | --- | --- | --- | --- |
| **Centrosome** | | | | | | | | | | | |
| Wang 2009 | Yes | *Neocortex of mouse embryos*  Mouse embryos at embryonic day E13.5 | yes | Mice used were CD-1 strain. | In utero electroporation was performed at 13.5 days gestation on pregnant CD-1 mice. Electroporated with Kaede-Centrin1. Brains were sliced, immunostained and followed by time lapse confocal microscopy. Photo conversion of Kaede is accompanied by a colour change from green to red. After one cell cycle the centrioles are red/green, after two cell cycles the mother centriole is red/green the daughters become all green. | NA | NA | NA | NR | **Low** | All procedures for animal handling and usage were approved by the institutional research animal resource centre. |
| Tamura 2001 | NR | *Oocytes of the starfish Asterina pectinifera were used throughout the study.* | no | To obtain follicle-free immature oocytes arrested at the germinal vesicle stage, isolated ovaries were treated with Ca-free artificial seawater and then transferred into seawater to induce spawning. | Oocytes were treated with 1 uM 1-methyladenine to induce meiosis, stained with fluorescent immunochemistry and analysed by fluorescent microscopy. | NA | NA | NA | NR | **Low** |  |
| Holy 1991 | NR | *8 cell embryos of sea urchins*  *Eggs and sperm from the sea urchins Lytechinus pictus and Strongylocentrotus purpuratus were used to artificially produce embryos.* | no | Embryos were cultured in suspension in artificial seawater with stirring until fourth cleavage. | Immunostained and observed by confocal fluorescent microscopy. | NA | NA | NA | NR | **Low** |  |
| Conduit 2010 | NR | *Drosophila neural stem cells*  Fly stocks: 2X GFP-PACT and 1X RFP-Cnn in a cnn null background under the control of the Ubq promoter. | yes | NR | 3rd instar larval brains were dissected and semi-squashed in PBS under a coverslip. Mother centrioles are segregated are the oldest (and therefore brightest) centrioles. | NA | NA | NA | NR | **Low** |  |
| Januschke 2011 | NR | *Drosophila neural stem cells*  NR | yes | NR | Fusion constructs were subcloned into the Drosophila and transgenic lines expressing PACT–d2Eos, mKATE-ASL and YFP–CNB under the control of the poly-Ubiquitin promoter were obtained using standard techniques. Primary cultures were derived from brains. Photoconversion was carried out and observed by 4D time lapse confocal. Photo-convertible EosFP56 protein activated by laser from green to red. Activation of the protein can occur before or after centriole duplication to track mother or daughter. | NA | NA | NA | NR | **UNR** | No description of model nor its routine maintenance |
| Yamashita 2007 | NR | *Drosophila male germline stem cells.*  *Fly strains: Heatshock(hs)-gal4 (Flybase), NGT40, cnnHK21 were obtained from Bloomington Drosophila Stock Center.* | yes | All Drosophila stocks were raised on molasses medium. | UAS-EGFP-PACT/hs-Gal4 flies were subjected to heat shock for 2.5 hours. Testes were then dissected and subjected to immunostaining. Both the mother and daughter centrosomes are labelled by GFP PACT in the first cell cycle after heat shock. In the second cell cycle, the daughter centrosome retains GFP-PACT, whereas the mother centrosome is not labelled. Strategy to label the mother centrosome: GFPPACT expressed during early embryogenesis using the NGT40 driver marks the centrioles (1 and 2) retained in the mother centrosomes after the depletion of cytoplasmic GFP-PACT | NA | NA | NA | NR | **Low** |  |
| Rusan 2007 | NR | *Drosophila neural stem cells*  y w flies were the wild-type controls. For live-cell imaging the following strains: UAS-actin-GFP (Jacinto et al., 2000), UAS-GFP-Cnn1 (Megraw et al., 2002). | yes | NR | Whole brains were dissected and imaged. | NA | NA | NA | NR | **Low** |  |
| Shimizu 1996 | NR | *Fertilized eggs and embryos of the freshwater oligochaete Tubifex hattai*  *were obtained as previously described (Shimizu 1982). For the experiments, all eggs and embryos were freed from their cocoons.* | no | NR | Embryos were maintained T 19-20°C. Whole-mount labelling during the first cleavage and during the second and subsequent cleavages | NA | NA | NA | NR | **Low** |  |
| **Centrosome (cilia)** | | | | | | | | | | | |
| Anderson 2009 | NR | *NIH 3T3 cells*  (American Type Culture Collection) | yes | NIH 3T3 cells were grown in Dulbecco’s modified Eagle medium (DMEM) + 10% normal calf serum (Invitrogen). | NIH 3T3 lines stably expressing GFP-centrin 2 were generated by transfection. myc-tagged Musmusculus a-tubulin isoform 2 (pTS488) was prepared as described elsewhere, NIH 3T3 cells were pulse labelled, cells were extracted to preferentially depolymerize cytoplasmic microtubules, fixed and processed for immunofluorescence. After transfection, myc-tagged a-tubulin incorporates into new centrioles such that, in G2, each of the two centrosomes has an unlabelled mother centriole and a labelled daughter centriole (green). After duplication and segregation in the second mitosis, there are sister cell pairs in which the mother centriole of one cell is unlabeled (the older mother centriole) and the mother centriole of the other cell is labelled (the newer mother centriole). | NA | no | NA | NR | **UNR** | Cell line which was not checked for contaminants |
| Piotrowska-Nitsche 2012 | Yes | *Neuroepithelium of mouse embryos*  Mouse embryos at embryonic day E7.5 and E8.5 | yes | Mice used were: Z/RED line (STOCK Tg(CAG-Bgeo,-DsRed*MST) 1Nagy/J, Jackson Laboratory, Bar Harbor, ME USA), CAGGCreER™ and the modified bacterial artificial chromosome (BAC) transgenic Olig2-enhanced GFP (eGFP) line (STOCK Tg (Olig2-EGFP) EK23Gsat/Mmcd, MMRRC). | Neural tube was dissected and sliced live imaging and monitoring of cilia formation. | NA | NA | NA | NR | **Low** | Mice were cared for according to animal protocols approved by Emory University. |
|  | NR | *IMCD3 mouse kidney cell line*  IMCD3, stably expressing somatostatin receptor 3 (SsTR3)-GFP in cilia (a kind gift from Greg Pazour) | yes | Grown in DMEM high glucose media without serum at 37°C in 5% CO2. | After 7.5 hours of serum starvation, cells were cultured in media with 10% FBS. Live cell imaging of division observed with confocal | NA | no | NA | NR | **UNR** | Cell line which was not checked for contaminants |
| **Endoplasmic Reticulum** | | | | | | | | | | | |
| Smyth 2015 | NR | *Drosophila neural stem cells*  Flytrap line: CC00735 (GFP-Sec61a). | yes | NR | Whole third-instar larval Drosophila brains or testes were dissected from third-instar larvae in Drosophila Schneider’s medium (Life Technologies) containing Antibiotic-Antimycotic (Life Technologies) and were mounted in the same medium for imaging. | NA | NA | NA | NR | **Low** |  |
| **Endosome** | | | | | | | | | | | |
| Beckmann 2007 | Yes | *CD34+ cells derived from human cord blood* | no | Human umbilical cord blood of G-CSF–treated stem cell donors were obtained from unrelated donors. CD34+ cells were isolated by magnetic cell separation using the MidiMacs technique. If not stained immediately, freshly purified CD34+ were cultured in at 37°C and 5% CO_2_ at a density of approximately 1x 10^5^ cells/ml. | CD34+ cells were culture in the presence of early-acting cytokines in I20 medium. To conserve their morphology, the CD34+ cells were prefixed for 5 minutes at room temperature with paraformaldehyde at a concentration of 0.2% in the medium. Fixed, immunostained and imaged. | P | P | yes | P | **Low** | Stem cells confirmed by CD34 and functional assays  Informed consent was obtained in accordance with the Declaration of Helsinki. |
| Coumailleau 2009 | NR | *Drosophila neural stem cells*  Temperature-sensitive Gal80ts, neu-Gal4, UAS-Sara-GFP, UAS-Pon-RFP, UAS-His-RFP, UAS-Rab5-GFP; UAS-2xFYVE-GFP1 stocks | yes | Gal80ts stocks were grown at 22°C until puparium formation, at which time the temperature was raised to the value specified for each experiment. | SARA-GFP endosomes or Rab5–GFP or PtdIns(3)P containing endosomes labelled with a FYVE–GFP11 probe and Pon-RFP are tracked through mitosis and imaged | NA | NA | NA | NR | **Low** |  |
| Emery 2005 | NR | *Drosophila external sensory* organs  NR | yes | NR | ES organs dissected from various Drosophila fly strain pupae transgenic for Rab proteins. Rab5-GFP (early endosomes), Rab7-GFP (late endosomes), and Rab11-GFP (recycling endosomes) fusion proteins are expressed in SOP cells using neuralized-Gal4 and visualized through a spinning disk confocal microscope. For the visualization of Sara endosomes, 100- 200 pg of venus sara, CFP sara or mRFP sara mRNA were injected into one-cell stage or into one cell of 16-32 cell stage wild-type or morphant embryos, and monitored by 3D confocal microscopy imaging in the neuroepithelium. | NA | NA | NA | NR | **UNR** | No description of model nor its routine maintenance, not clear how venus SARA or YFP Rab genes generated |
| Kressmann 2015 | NR | *Zebrafish neural tube*  NR | no | Zebrafish strains were grown at 28°C and manipulated in Danieau embryo medium. | Lineage tracing was carried out by single-cell injection of RNA encoding the photoconvertible protein Kaede5 in 32-cell stage embryos. For the visualization of Sara endosomes, 100 200 pg of venus sara, CFP sara or mRFP sara mRNA were injected into one cell of 16/32-cell stage wild-type or morphant embryos, and monitored by 3D confocal microscopy imaging in the neuroepithelium at the corresponding developmental stage. For the co-localization experiments of Sara to early, recycling and late endosomes, 100-200 pg of the CFP rab5c, eCFP rab7 or eYFP- rab11a mRNAs were injected. | NA | NA | NA | NR | **UNR** | No details regarding fish |
| Loubery 2014 | NR | *Drosophila external sensory organ precursor (SOP)*  Fly strains: w; UAS Sara-GFP, w: UAS-Rab11-GFP, UAS-Rab7-GFP, UAS-Pon-RFP,  UAS-tubGal80ts/+ (Control), tub-Gal80ts/+.UAS-alpha tubulin-GFP, esg-Gal4, UAS-mCherry-Tubulin | yes | Fly strains were kept at 16 C until puparium or at room temperature for the MARCM expts. | Nota were dissected and immunostained or imaged | NA | NA | NA | NR | **Low** |  |
| Montagne 2014 | NR | *Primary tissue culture of the Drosophila adult midgut*  Female flies were used for all experiments. Fly stocks: esg-Gal4, tub-GAL80, UAS-Sara GFP, UAS-Pon-RFP. Adult midguts were used. | yes | Drosophila stocks were maintained at 18-21°C on standard fly medium. 4.5 days after hatching progeny were shifted to 29°C for 13 hr. | Flies were infected with bacteria to increase numbers of ISC. The midgut was dissected and placed into tissue culture or chamber slide. Fixed, immunostained and observed by spinning disc microscopy. | No | NA | NA | NR | **High** | No details for stem cell characteristics, nor how these are characterised for asymmetric results. |
| **Midbody** | | | | | | | | | | | |
| Kuo 2014 | NR | *hRPE-1, human retinal epithelial cell line*  Purchased from Clontech | no | NR | Centrin was GFP tagged and then mid body was immunostained with MLKP1 | NA | no | NA | NR | **UNR** | No description of model nor its routine maintenance |
|  | NR | *H1 (WA01) human embryonic stem cell line*  NR.  Cells were used within 10 passages. | no | NR | Centrin was GFP tagged and then mid body was immunostained with MLKP1 | NA | no | NA | NR | **UNR** | No description of model nor its routine maintenance |
| Goss 2008 | NR | *BSC-1 monkey epithelial kidney*  NR | no | BSC1 cells were grown in DME supplemented with 10% FBS, 1% penicillin/ streptomycin, 1% L- glutamine, and 1% nonessential amino acids (Invitrogen). | Cells were cultured at 37 °C supplemented with 5% CO 2, and plated on dishes (Mattek) before imaging. BSC1 cells were fixed and immunostained with αMKLP1. | NA | no | NA | NR | **UNR** | Unclear model details. |
| **Midbody; centrosome** | | | | | | | | | | | |
| Salzmann 2013 | NR | *Drosophila male and female germline stem cells.*  Ubi-Pavarotti-GFP (Minestrini et al., 2002; obtained from David Glover, University of Cambridge); Ubi-Cnb-YFP (Januschke et al., 2011; obtained from Cayetano Gonzalez, IRB Barcelona) | yes | All fly stocks were raised in standard Bloomington medium at 25°C. | Green, Pavarotti-GFP (Pav-GFP) marking MRs and ring canals were observed by time lapse microscopy. Centrosomes were marked with Cnb-YFP | NA | NA | NA | NR | **Low** |  |
| **Mitochondria** | | | | | | | | | | | |
| Rivolta 2002 | NR | *UB/UE-1 (a bipotent progenitor that is able to produce neonatal hair cells as well as supporting cells upon differentiation)*  NR | no | Routinely grown on uncoated tissue culture plastic in the presence of MEM, 10% fetal calf serum and 50 U/ml of g-interferon at 33°C. | Differentiation was induced 1–2 days after seeding at 33^o^C by replacing the media with without g-IFN and then transferring to 39^o^C. Cells were allowed to differentiate for 1, 2 and 14 days. MitoTracker Red used to stain mitochondria.  To monitor the fate of aged organelles, we expressed paGFP in mitochondria, by fusing the fluorescent protein omp25. paGFP fluoresces after exposure to a pulse of ultraviolet (UV) light. Because synthesis of paGFP continues after the light pulse, cells subsequently accumulate unlabeled “young” components in addition to the labelled “old” components | NA | no | NA | NR | **UNR** | No model details, or checking that the cell lines had no contaminants |
|  | NR | *UB/OC-1 (a conditionally immortal, unipotent population of committed, cochlear hair cell precursors)*  NR | no |  |  | NA | no | NA | NR | **UNR** | No model details, or checking that the cell lines had no contaminants |
| **Mitochondria; endoplasmic reticulum** | | | | | | | | | | | |
| Dalton 2013 | NR | *Germinal vesicle stage oocytes from 4-6 week old mice*  Female MF1 mice | no | Oocytes placed in M2 medium at 37°C | Germinal vesicle arrest was maintained with IBMX, release from arrest was achieved though washing, 7% ethanol was used to activate polar body extrusion. Oocytes microinjected with mito-GFP/ Dil 18 to visualise mitochondria/ dsRed2ER for ER. Fixed after meiotic arrest and fluorescence quantified with confocal. | NA | NA | NA | NR | **Low** |  |
| **Mitochondria; lysosome; golgi** | | | | | | | | | | | |
| Katajisto 2015 | NR | *Human mammary epithelium - stem like*  FACS enriched primary mammary culture FL2+, CD44hi | no | Cells were maintained in MEGM Mammary Epithelial Cell Growth Medium (Lonza) or CnT-27 PCT Mammary Epithelium Medium (CELLnTECH Advanced Cell Systems). | Lentiviral expression of pa GFP fusion proteins were generated by PCR cloning. UV laser activates the pa-GFP; subsequent organelle formed is non-fluorescent (unactivated). To monitor the fate of aged organelles, paGFP fluoresces only after exposure to a pulse of UV-light. Because synthesis of paGFP continues after the light pulse, cells subsequently accumulate unlabeled ‘young’ components in addition to the labelled ‘old’ components. | No | NA | NA | NR | **High** | No details for stem cell ability besides mammosphere. Assumes mammosphere formation is consistent with stem cell properties |
| **P granule** | | | | | | | | | | | |
| Gallo 2010 | NR | *4 cell embryo of C. Elegans*  C. Elegans strains (pie-1 prom::GFP::PGL-1-pgl-1 3ʼUTR) were derived from the wild-type Bristol N2 | yes | C. Elegans strains (pie-1 prom::GFP::PGL-1-pgl-1 3ʼUTR) derived from the wild-type Bristol N2 and reared with standard procedures. All experiments performed at 24^o^C. | Confocal time-lapse images of zygotes expressing GFP::PGL-1. | NA | NA | NA | NR | **Low** |  |
| Rose 1998 | NR | *2-4 cell embryo of C. Elegans*  Strains were obtained from the Caenorhabditis Genetics Centre or constructed in the Kemphues laboratory. For all experiments, worms were grown at 20°C, but during videomicroscopy, embryos were at 23–25°C. | no | C. Elegans unc-22(e66) strains were cultured as described in Brenner (1974). | Embryos were cut from hemaphrodites, immunostained and filmed using time-lapse video recorder. | NA | NA | NA | NR | **Low** |  |
| Boyd 1996 | NR | *2 cell embryo of C. Elegans*  Bristol N2 strain | no | NR | Embryos were cut from hemaphrodites and immunostained. | NA | NA | NA | NR | **Low** |  |
| Pang 2004 | NR | *C. Elegans embryo up to 4 cell stage*  The Bristol strain N2 was used as the standard wildtype strain. | no | C. Elegans culture and genetics were as described (Brenner, 1974). | Immunostained for p granules. | NA | NA | NA | NR | **Low** |  |
| **Proteosome** | | | | | | | | | | | |
| Ogrodnik 2014 | NR | *CHO (Chinese hamster ovary), HEK (human embryonic kidney) cell lines*  NR | no | Cells were cultured in high-glucose DMEM supplemented with FBS, glutamine and antibiotics. | Cells were transiently transfected with Dendra-VHL. Localization of active proteasomes was assessed by measuring the increase of blue-channel fluorescence after addition of fluorogenic proteasome substrate. | NA | no | NA | NR | **UNR** | No model details. Unclear which cell line was used for asymmetry results (Fig 2A) |
| Chang 2011 | Yes | *T cells*  CD4+ or CD8+T cells from WT C57BL/6 mice, no further details. | no | All mice were housed in pathogen free conditions prior to use. | T cells from WT C57BL/6 activated to induce T cell division, followed by immunostaining | NA | NA | yes | NR | **Low** | Mice were cared for according to animal protocols approved by Pennsylvania University |
| **Spectrosome/ fusome** | | | | | | | | | | | |
| de Cuevas 1998 | NR | *Drosophila germline cysts (oocyte)*  NR | no | Flies were maintained at 22-25˚C on standard medium; they were transferred to bottles containing wet yeast for 1-2 days before dissections. | Ovaries were triple-stained with anti-anillin antibodies (green), anti-hts antibodies (red) and DAPI (not shown). Imaged. | NA | NA | NA | NR | **UNR** | No reporting of model other than 'drosophila.' |
| Lin 1995 | NR | *Drosophila germline cysts (oocyte)*  Canton-S ry506 flies | no | Flies were raised at 22-25°C and were aged for 3-5 days on yeast containing corn meal/agar medium prior to examination | Ovaries were dissected for immunostaining. | NA | NA | NA | NR | **Low** |  |
| UNR= unclear or not reported; NR = not reported; U = unclear; NA = not applicable; pa = photoactivatable; GFP = green fluorescent protein; RFP = red fluorescent protein; YFP = yellow fluorescent protein; Cnb = centrobin; Cnn = centrosomin  Maternal organelle = original organelle, whilst daughter = newly synthesised organelle. | | | | | | | | | | | |
