# Supplementary material for: A systematic review of the asymmetric inheritance of cellular organelles in eukaryotes: A critique of basic science validity and imprecision
Source: PLoS One. 2017 May 31;12(5):e0178645. doi: 10.1371/journal.pone.0178645 (PMC5451095; doi:10.1371/journal.pone.0178645)
Supplement: S4 Table — (DOCX) [file pone.0178645.s006.docx]

**S4 Table. Marker validity tool**

| **Domain** | **Signalling question** | **Notes** | **Answer** |
| --- | --- | --- | --- |
| **Validation of Marker** | **1. Functional validation according to report aims or methods.** |  | Free text |
|  | **2. Cellular localisation according to Genecard confidence 5 or cellular components according to Flybase** | [http://www.genecards.org/ http://flybase.org/](http://www.genecards.org/%20http://flybase.org/) | Free text  or NA/NR |
|  | **3. gene ontology - cellular component terms according to Genecards** | <http://www.genecards.org/> | Free text  or NA/NR |
|  | **4. Do the authors present data for functional validation in results?** | This includes:  Is the marker in the correct location?  Any functional experiments? | Yes/ NR/Referenced  If yes add free text to justification. |
|  | **5. Were co-localisation experiments performed with a second marker/ was the result confirmed with a second marker?** |  |  |
|  | **Validation rating** | Low= no concerns.  Unclear/not reported = insufficient data to make a judgement or not reported  High risk = there are concerns | **Low/UNR/High/**  **Referenced**  **If UNR/High add free text to justification** |
| **Controls** | **6. Is there an appropriate positive control?** | Molecular: Result in the presence of another tagged protein/gene that marks the organelle of interest.    IHC:  Result in another model that expresses the marker | Yes/NR/NA  If yes add free text to justification. |
|  | **7. Is there an appropriate negative control?** | Molecular: Result in the presence of a tagged protein that does not mark the organelle of interest OR in the absence of a tagged protein (e.g. empty vector, tag only).   IHC: Result in absence of marker, AND result in another model than does not express the marker | Yes/NR/NA  If yes add free text to justification. |
|  | **Control rating** | Low= no concerns.  Unclear/not reported = insufficient data to make a judgement or not reported.  High risk = there are concerns | **Low/UNR/High**  **If UNR/High add free text to justification** |
| **Experimental performance, reporting flaws** | **8. Were there sufficient details to judge the performance of molecular experiments?** | Detailed =, allowing repetition of the experiment.  Partial = some details, but could not repeat the experiment easily.  NR = not reported | D/P/NR/NA |
|  | **9. Did the authors provide evidence that the genetic manipulation did not influence the observed effect?** |  | Yes/NR/NA  If yes add free text to justification. |
|  | **10. Molecular techniques: Additional comments/ concerns** |  | Yes/NR/NA  If yes add free text to justification. |
|  | **11. Were there sufficient details to judge the performance of immunochemistry?** | Detailed = allowing easy repetition of the experiment.  Partial = some details, but could not repeat the experiment easily.  NR = not reported | D/P/NR/NA |
|  | **12. Immunotechniques: Additional comments/ concerns** |  | Yes/NR/NA  If yes add free text to justification. |
|  | **13. Type of image analysis.** | Confocal fluorescent / fluorescent  NR/ light |  |
|  | **14. Were there sufficient details to repeat the image analysis?** | Detailed =, allowing easy repetition of the experiment.  Partial = some details, but could not repeat the experiment easily. NR = not reported | D/P/NR/NA |
|  | **15. Was the optical plane considered?** |  | Yes/NR/NA |
|  | **16. Additional comments/concerns regarding imaging** |  | Yes/NR/NA  If yes add free text to justification. |
|  | **Experimental performance rating** | Low= no concerns.  Unclear/not reported = insufficient data to make a judgement or not reported.  High risk = there are concerns | **Low/UNR/High**  **If UNR/High add free text to justification** |
| **Applicability/ generalisability** | **Model variability**  **(Did the experiment give the same result in a different model?)** | Yes = low  NR = UNR | **Low/UNR**  **If low add free text to justification** |
| **Additional Biases** | **17. Any experimental assumptions?** |  | Yes/NR/NA  If yes add free text to justification. |
|  | **18. Other concerns/**  **How was asymmetry measured?** | Was subjective assessment used, if yes the results should be verified independently | Yes/NR/NA  If yes add free text to justification. |
|  | **19. Was the marker stated a priori?** | The marker should be stated a priori in the introduction or methods. Or the authors should assess a range of markers clearly stated in the aims. If the authors list the marker in the methods or results only (with no further details or intention) this is unclear/NR. | Yes/NR/No |
|  | **additional rating** | Low= no concerns.  Unclear/not reported = insufficient data to make a judgement or not reported.  High risk = there are concerns | **Low/UNR/High**  **If UNR/High add free text to justification** |
| **OVERALL RATING** | | Low= all domains clearly reported. Unclear = Any domains are unclear, but not high risk. High risk = there is a concern of high risk | **Low/UNR/High** |
| **JUSTIFICATION** | | Free text to explain UNR or High ratings, plus additional free text from signalling questions | |

Note that if several overall ratings inform one asymmetry result (if there is an organelle marker and a cell specific marker) then a second overall judgement is made based on the same instructions notes for the overall rating.

**Model Validity Tool Assessments**

|  | **Organelle or niche marker** | **1.** | **2.** | **3.** | **4.** | **5.** | **Marker**  **Validation rating** | **6. positive** | **7. negative** | **Control rating** |
| --- | --- | --- | --- | --- | --- | --- | --- | --- | --- | --- |
| ***Centrosome*** | | | | | | | | | | |
| Conduit 2010 | Centrobin RFP | Drosophila homologue of the human, daughter centriole marker | centriole; centrosome; ciliary basal body; pericentriolar material | NA | Yes |  | **Low** | NR | NR | **UNR** |
|  | PACT GFP | PACT is a Drosophila centriolar protein | Centriole | NA | Yes |  | **Low** | NR | NR | **UNR** |
| Holy 1991 | anti-4D2 | antibody to centrosomal material | NA | NA | Yes | Yes | **Low** | NR | NR | **UNR** |
| Januschke 2011 | PACT–d2Eos | Pancentriolar marker according to text. | Centriole | NA | Yes |  | **Low** | NR | NR | **UNR** |
|  | ASL-mKATE | Drosophila homologue of the human, daughter centriole marker | centriole; centrosome; ciliary basal body; pericentriolar material | NA | Yes |  | **Low** | NR | NR | **UNR** |
|  | Centrobin-YFB | Drosophila homologue of the human, daughter centriole marker | Centriole | NA | Yes. |  | **Low** | NR | NR | **UNR** |
| Saltzmann 2013 | Cnb-YFP | Centrobin Drosphila homolougue of human daughter centriole marker | Centriole | NA | Yes |  | **Low** | NR | NR | **UNR** |
| Shimizu 1996 | ɣ tubulin | Stains centrosomes in vertebrate cells, (referenced). | cytoskeleton; endosome; cytosol; nucleus(TUBG1) | pericentriolar material; condensed nuclear chromosome; gamma-tubulin complex; cytoplasm; centrosome | Yes |  | **Low** | NR | NR | **UNR** |
| Tamura 2001 | Phase microscopy of nucleus/ aster | NA | NA | NA | NR |  | **NA** | NA | NA | **NA** |
| Wang 2009 | Centrin1-Kaede | A central component of the centriole | cytoskeleton | spindle pole; centrosome; centriole; photoreceptor connecting cilium | Yes. |  | **Low** | NR | NR | **UNR** |
|  | Pax6 | A radial glial progenitor marker | nucleus | nuclear chromatin; intracellular; nucleus; nucleoplasm; cytoplasm | NR |  | **UNR** | NR | NR | **UNR** |
|  | TUJ1 | A differentiating neuronal marker | cytoskeleton; extracellular; nucleus | cytoplasm; microtubule; axon; dendrite | NR |  | **UNR** | NR | NR | **UNR** |
| Yamashita 2007 | Fas III (IHS) | NR. Appears to be a marker of Hub (niche) | cytoplasm; cytosol; neuromuscular junction; nuclear membrane; plasma membrane; recycling endosome; smooth septate junction | NA | NR |  | **UNR** | NR | NR | **UNR** |
|  | GFP PACT | PACT is a Drosophila centriolar protein | Centriole | NA | Yes  Ref. |  | **Low** | NR | NR | **UNR** |
|  | Vasa (IHS) | In Drosophila, vasa expression is seen in germ cells | cytoplasm; cytoplasmic ribonucleoprotein granule; P granule; perinuclear region of cytoplasm; pole plasm; spectrosome | NA | Ref. |  | **Ref** | NR | NR | **UNR** |
| Rusan 2007 | Cnn-GFP | We imaged centrosomes using a PCM protein, GFP-Cnn (Megraw et al., 2002) | Centriole; centrosome; pericentriolar material; spindle pole | NA | Yes  Ref. |  | **Low** | NR | NR | **UNR** |
| ***Centrosome (cilia)*** | | | | | | | | | | |
| Anderson 2009 | Centrin 2 GFP | centriole/ cilia | cytoskeleton | photoreceptor connecting cilium; ciliary transition zone; ciliary basal body | Yes  Ref. |  | **Low** | NR | NR | **UNR** |
|  | α tubulin | centriole/ cilia | cytoskeleton; extracellular (TUBA1A) | nucleus; cytosol; microtubule; cytoplasmic microtubule; cytoplasmic ribonucleoprotein granule (TUBA1A) | Yes.  Ref. |  | **Low** | NR | NR | **UNR** |
|  | α tubulin- myc | centriole/ cilia | cytoskeleton; extracellular (TUBA1A) | nucleus; cytosol; microtubule; cytoplasmic microtubule; cytoplasmic ribonucleoprotein granule (TUBA1A) | Yes.  Ref. |  | **Low** | NR | NR | **UNR** |
| Piotrowska-Nitsche 2012 | Sstr3 GFP | Somatostatin receptor 3 is a marker of the ciliary membrane | None at 5 | cytoplasm; plasma membrane; integral component of plasma membrane; cilium; integral component of membrane | Yes.  Ref. |  | **Low** | NR | NR | **UNR** |
|  | Sstr3 GFP virus | Somatostatin receptor 3 is a marker of the ciliary membrane | None at 5 | cytoplasm; plasma membrane; integral component of plasma membrane; cilium; integral component of membrane | Yes.  Ref. |  | **Low** | NR | NR | **UNR** |
| ***Endoplasmic Reticulum*** | | | | | | | | | | |
| Dalton 2013 | DiI 18 | NR. Lipophilic tracer for ER | NA | NA | No |  | **Low** | no | no | **UNR** |
| Smyth 2015 | Baz | Marker for apical cell/neuroblast (no reference) | adherens junction; apical junction complex; apical plasma membrane; spot adherens junction; zonula adherens | NA | Yes. |  | **Low** | no | no | **UNR** |
|  | Sec61 α GFP | ER membranes (no reference) | endomembrane system; endoplasmic reticulum; fusome; rough endoplasmic reticulum | NA | Yes |  | **Low** | no | no | **UNR** |
| ***Endosome*** | | | | | | | | | | |
| Beckmann 2007 | CD53 | None. Chosen as a protein that is asymmetrically divided, but referenced as associated with endosomes. | extracellular; plasma membrane | Immunological synapse; plasma membrane; integral component of plasma membrane; cell-cell junction; cell surface | U |  | **UNR** | no | no | **UNR** |
|  | CD63 | None. As above. | endosome; vacuole; lysosome | Extracellular space; lysosome; lysosomal membrane; late endosome; plasma membrane | U |  | **UNR** | no | no | **UNR** |
|  | CD71 | None. As above. | endosome | Extracellular region; extracellular space; mitochondrion; endosome; plasma membrane. | U |  | **UNR** | no | no | **UNR** |
| Coumailleau 2009 | FYVE GFP | FYVE associated with early endosomes and other PI(3)P containing vesicles | NR | NA | U  Ref |  | **Ref** | no | no | **UNR** |
|  | Pon RFP | Pon labels nuclear membrane and cortex of pIIb | cell cortex; cytoplasm | NA | Yes.  Ref |  | **Low** | no | no | **UNR** |
|  | PtdIns(3)P GFP | phosphatidylinositol-3-phosphate endosome | NR | NA | No. |  | **High** | no | no | **UNR** |
|  | Rab5 GFP | Rab5 is present on early endosomes | early endosome; lipid particle; neuronal cell body; plasma membrane; synapse | NA | Ref. |  | **Low** | no | no | **UNR** |
|  | SARA GFP | SARA is present in endosomes | Early endosome | NA | Ref. |  | **Ref** | no | no | **UNR** |
| Emery 2005 | Pon RFP | Pon labels nuclear membrane and cortex of pIIb | Cell cortex; cytoplasm | NA | Ref |  | **Low** | no | no | **UNR** |
|  | Rab11 GFP | labels recycling endosomes | Autophagosome; cell pole;  Centrosome; cleavage furrow; cytoplasm; cytoplasmic vesicle; endosomes; fusome; Golgi lipid particle; microtubule organizing center; neuronal cell body; nuclear envelope; perinuclear; plasma membrane; recycling endosomes;  spindle envelope; synapse; terminal bouton; vesicle | NA | Yes | Yes | **Low** | yes | no | **UNR** |
|  | Rab5 GFP | labels early endosomes | early endosome; lipid particle; neuronal cell body; plasma membrane; synapse | NA | Ref |  | **Low** | no | no | **UNR** |
|  | Rab7 GFP | labels late endosomes | late endosomes; late endosome membrane; lipid particle; neuronal cell body; plasma membrane; synapse | NA | Ref |  | **Low** | no | no | **UNR** |
| Kressman 2015 | Rab11a YFP | component of recycling endosomes | cytoskeleton, cytosol, endosome, extracellular, golgi | spindle pole; cytoplasm;  mitochondrion; multivesicular body; Golgi apparatus | No. | No. | **UNR** | no | no | **UNR** |
|  | Rab5c YFP | component of early endosomes | extracellular, lysosome, vacuole | early endosome, early endosome membrane, melanosome, extracellular exosome | No. | No. | **Low** | no | no | **UNR** |
|  | Rab7 YFP | component of late endosomes | endosome, extracellular, lysosome, vacuole | autophagosome membrane; cytoplasm; colocalizes with lysosome; lysosomal membrane; colocalizes with late endosome | No. | No. | **Low** | no | no | **UNR** |
|  | Sara Venus | labels apical early endosomes | endosome, cytosol, extracellular, plasma membrane | Early endosome, membrane. cytosol | No. | No. | **High** | yes | no | **UNR** |
| Loubery 2014 | Pon RFP | Pon labels nuclear membrane and cortex of pIIb | cell cortex; basal cortex; cytoplasm | NA | Yes. |  | **Low** | no | no | **UNR** |
|  | Sara GFP | labels early endosomes | Early endosome | NA | U |  | **UNR** | no | no | **UNR** |
| Montagne 2014 | PH3 IHS | NR. Appears to be a marker for ISCs | nucleosome | NA | No |  | **UNR** | no | no | **UNR** |
|  | Pon RFP | Pon labels nuclear membrane and cortex of pIIb | cell cortex; basal cortex; cytoplasm | NA | Yes |  | **Low** | no | no | **UNR** |
|  | Rab 11 GFP | recycling endosome | cytoskeleton, cytosol, endosome, extracellular, golgi | NA | U |  | **UNR** | no | no | **UNR** |
|  | Rab 7 GFP | late endosome | late endosome; late endosome membrane; lipid particle; neuronal cell body; plasma membrane; synapse | NA | Yes. |  | **Low** | no | no | **UNR** |
|  | SARA RFP | labels early endosomes | Early endosome | NA | U |  | **UNR** | no | no | **UNR** |
|  | α tubulin mcherry | centriole/ cilia | cytoskeleton, extracellular | NA | Yes |  | **Low** | no | no | **UNR** |
| ***Golgi*** | | | | | | | | | | |
| Katajisto 2015 | 1,4-galactosyltransferase (81 N-term aa) paGFP | NR | extracellular; golgi | membrane | No |  | **UNR** | no | no | **UNR** |
| ***Lysosome*** | | | | | | | | | | |
| Katajisto 2015 | lamp2 pa GFP | NR | endosome; extracellular; lysosome; plasma membrane; vacuole | lysosome; lysosomal membrane; membrane; platelet dense granule membrane; lysosomal lumen | No |  | **UNR** | no | no | **UNR** |
| ***Midbody*** | | | | | | | | | | |
| Goss 2008 | αMKLP1 | Midbody marker protein MKLP1 (Mishima et al., 2002) | cytoskeleton; nucleus | Nucleus; nucleoplasm; centrosome; spindle; cytosol | Yes |  | **Low** | no | no | **UNR** |
| Kuo 2014 | CETN1-GFP | Centrin 1 centrosome marker | cytoskeleton | spindle pole; centrosome; centriole; photoreceptor connecting cilium | Ref |  | **Ref** | no | no | **UNR** |
|  | MKLP1 | midbody marker | cytoskeleton; nucleus | Nucleus; nucleoplasm; centrosome; spindle; cytosol | Ref |  | **Ref** | no | no | **UNR** |
|  | α tubulin | To reflect the cell-stage-specific chromosomal morphology and microtubule organization' | astral microtubule; centrosome; cytoplasm; nucleus; perinuclear region of cytoplasm; spindle | NR | Yes  Ref |  | **Low** | no | no | **UNR** |
| Saltzmann 2013 | Add | The spectrosome, a germline-specific membranous organelle marked by Hts/adducin-like (Add) | actin filament; cytoplasm; fusome; germline ring canal; lateral plasma membrane; lipid particle; plasma membrane; spectrosome | NA | Ref. |  | **Ref** | no | no | **UNR** |
|  | Fas III | FasIII (fasciclin III) marks hub cells | cytoplasm; cytosol; neuromuscular junction; nuclear membrane; plasma membrane; recycling endosome; smooth septate junction | NA | Ref |  | **Ref** | no | no | **UNR** |
|  | GFP Pavarotti | Pavarotti is a homologue of MKLP1, a kinesin-like protein that is required for cytokinesis (Adams et al., 1998; Minestrini et al., 2002, 2003). | axoneme; cleavage furrow; contractile ring; female germline ring canal; kinetochore microtubule; male germline ring canal; midbody; mitotic spindle midzone; nucleus; somatic ring canal | NA | Yes |  | **Low** | no | no | **UNR** |
|  | Spd-2 | NR | centriole; centrosome; pericentriolar material | NA | Yes |  | **Low** | no | no | **UNR** |
|  | Vasa | In Drosophila, vasa expression is seen in germ cells | cytoplasm; cytoplasmic ribonucleoprotein granule; P granule; perinuclear region of cytoplasm; pole plasm; spectrosome | NA | Ref |  | **Ref** | no | no | **UNR** |
|  | γ-tubulin | NR | cytoplasma; gamma-tubulin complex; gamma-tubulin large complex; gamma-tubulin ring complex; gamma-tubulin small complex; microtubule; pericentriolar material; spindle | NA | Yes |  | **Low** | no | no | **UNR** |
| ***Mitochondria*** | | | | | | | | | | |
| Katajisto 2015 | Omp25 paGFP | NR. mitochondrial outer membrane protein 25 | mitochondrion | mitochondrion; mitochondrial outer membrane | NR | Yes | **Low** | no | no | **UNR** |
| Rivolta 2002 | Mab48 | labels mortalin, mitochondrial protein | extracellular; mitochondrion | cytoplasm; focal adhesion; extracellular exosome | NR | Yes | **Low** | no | no | **UNR** |
| Dalton 2013 | cytochrome c | NR | Cytosol; mitochondrion; nucleus | mitochondrion; mitochondrial inner membrane | No |  | **Low** | no | no | **UNR** |
|  | mito-GFP | NR. Used to localise mitochondria (an expression vector fused to a mitochondrial targeting sequence) | NA | NA | No |  | **Low** | no | no | **UNR** |
|  | TOM20 | NR | mitochondrion | mitochondrial outer membrane translocase complex | No |  | **Low** | no | no | **UNR** |
| ***P granule*** | | | | | | | | | | |
| Gallo 2010 | PGL1 GFP | P granule protein PGL-1 (referenced Kawasaki 1998) | NA | NA | NR | Yes | **Low** | yes | no | **UNR** |
| Rose 1998 | Ab OIC1D4 or K76 | None. Reference provided for Strome and Wood. | NA | NA | Ref |  | **Ref** | yes | no | **UNR** |
| Boyd 1996 | Ab OIC1D4 | None. Reference provided for Strome and Wood. | NA | NA | Ref |  | **Ref** | no | no | **UNR** |
| Pang 2004 | K76 | P granule marker | NA | NA | Yes. |  | **UNR** | no | no | **UNR** |
| ***Proteosome*** | | | | | | | | | | |
| Chang 2011 | proteosome 20s alpha 1 | component of the proteosome | cytoskeleton, cytosol, extracellular, nucleus | centrosome, extracellular endosome | NR | Yes. | **Low** | yes | no | **UNR** |
| Ogrodnik 2014 | mito-dendra 2 | labels mitochondria (subunit VIII of cytochrome C) | mitochondrion | mitochondrion respiratory chain complex IV | Yes. |  | **Low** | NA | UNR | **UNR** |
|  | pDendra2-VHL | von Hippen-Lindau protein is a misfolded protein used to identify JUNQ, (assuming JUNQ are degradation centres) | cytosol, endoplasmic reticulum, mitochondria, nucleus | cytosol | Yes. |  | **Low** | UNR | UNR | **UNR** |
| ***Spectrosome/ fusome*** | | | | | | | | | | |
| de Cuevas 1998 | anillin | Anillin is expressed in actively dividing cells, at telophase, it is highly enriched in the cleavage furrow (ref.) | cleavage furrow; contractile ring; cytoplasm; germline ring canal; male germline ring canal; nucleus | NA | Yes |  | **Low** | no | no | **UNR** |
|  | hts protein | hts marks the fusome. | actin filamen; cytoplasm; fusome; germline ring canal; lateral plasma membrane; lipid particle; plasma membrane; spectrosome | NA | Yes |  | **Low** | no | no | **UNR** |
| Lin 1995 | α spectrin | α -spectrin is localized in germline stem cells and cystoblasts within a large cytoplasmic sphere (herein termed the spectrosome). | basolateral plasma membrane; cell cortex; fusome; Golgi apparatus; lipid particle; neuromuscular junction; plasma membrane; spectrosome | NA | Yes |  | **Low** | no | no | **UNR** |
|  | α tubulin | To reflect cell-stage-specific chromosomal morphology and microtubule organization' | astral microtubule; centrosome; cytoplasm; nucleus; perinuclear region of cytoplasm; spindle | NA | Yes |  | **Low** | no | no | **UNR** |
| UNR= unclear or not reported; NR = not reported; U = unclear; NA = not applicable; Ref = referenced; pa = photoactivatable; GFP = green fluorescent protein; RFP = red fluorescent protein; YFP = yellow fluorescent protein; Cnb = centrobin; Cnn = centrosomin; IHS = immune  Maternal organelle = original organelle, whilst daughter = newly synthesised organelle. | | | | | | | | | | |

|  | **Organelle or niche marker** | **Experimental performance** | | | | | | | | | | **Generalisability** | **Additional rating** | | | | **OVERALL RATING** | |
| --- | --- | --- | --- | --- | --- | --- | --- | --- | --- | --- | --- | --- | --- | --- | --- | --- | --- | --- |
|  |  | **Molecular techniques** | | | **Immuno**  **techiniques** | | **Imaging techniques** | | | |  |  |  | | | | **rating** | **Justification** |
|  |  | **8.** | **8.** | **10.** | **11.** | **12.** | **13.** | **14.** | **15.** | **16.** | **rating** | **rating** | **17.** | **18.** | **19.** | **rating** |  |  |
| ***Centrosome*** | | | | | | | | | | | | | | | | | | |
| Conduit 2010 | Centrobin RFP | P | no | NR |  |  | Con Fl | D | yes |  | ***UNR*** | ***UNR*** | yes | Asymmetry based on qualitative visual inspection | yes | ***Low*** | ***UNR*** | No marker or genetic controls. Cellular location is accurate.  Based on mother centriole being bright. Asymmetry measure based on centriole age. |
|  | PACT GFP | P | no | NR |  |  | Con Fl | D | yes |  | ***UNR*** | ***UNR*** | yes |  | yes | ***Low*** | ***UNR*** |  |
| Holy 1991 | anti-4D2 |  |  |  | P | NR | Con Fl | D | yes |  | ***Low*** | ***UNR*** |  | Mean pixel value and area were measured per optical stack. Total amount of centrosomal material in animal blastomeres estimated by multiplying mean value of one spindle pole by 2; total centrosomal material in vegetal blastomeres obtained by adding macromere and micromere poles. | NR | ***Low*** | ***UNR*** | Antibodies Ah6 and 5051, gave similar results. No negative controls.  Cellular location is accurate. Asymmetry measure is clear |
| Januschke 2011 | PACT–d2Eos | P | no | NR |  |  | Fl | D | NA |  | ***UNR*** | ***UNR*** |  | Asymmetry based on qualitative visual inspection | NR | ***Low*** | ***UNR*** | No controls, no genetic control  Cellular location is accurate.  Assumes expected outcomes from photoconversion will be true (for centriole age). |
|  | ASL-mKATE | P | no | NR |  |  | Fl | D | NA |  | ***UNR*** | ***UNR*** |  |  | NR | ***Low*** | ***UNR*** |  |
|  | Centrobin-YFB | P | no | NR |  |  | Fl | D | NA |  | ***UNR*** | ***UNR*** |  |  | yes | ***Low*** | ***UNR*** |  |
| Saltzmann 2013 | Cnb-YFP | P | no | NR |  |  | Con Fl | L | UNR |  | ***UNR*** | ***UNR*** | Yes | Unclear, but asymmetry appears to be a qualitative visual inspection (yes or no), which is subsequently quantified for cell number. | NR | ***Low*** | ***UNR*** | No marker or genetic controls.  Assumes Cnb marks the daughter centriole.  Cellular location is accurate. Unclear if focal plane considered. |
| Shimizu 1996 | ɣ tubulin | NA | NA | NR | D | NR | light | NR | NA |  | ***Low*** | ***UNR*** |  | Asymmetry measure is unclear, appears to be visual inspection based on immunoreactive granular structures. | yes | ***Low*** | ***UNR*** | lack of controls.  Cellular location is accurate. Unclear asymmetry measure. |
| Tamura 2001 | Phase micro-scopy of nucleus/ aster | NA | NA | NR |  |  | light | P | NA |  | ***Low*** | ***UNR*** | Yes | Asymmetry measure is unclear, appears to be visual inspection | NA | ***UNR*** | ***UNR*** | No controls in absence of artificial activation of oocytes or transplantation of polar bodies. |
| Wang 2009 | Centrin1-Kaede | D | no | NR |  |  | NR | NA | UNR |  | ***UNR*** | ***UNR*** |  | Asymmetry measure is unclear, appears to be visual inspection | yes | ***Low*** | ***UNR*** | No marker or genetic controls and unclear if optical plane was considered or if this was important. Unclear marker validation for Pax 6 and TUJ1 (no references). |
|  | Pax6 IHS |  |  |  | P | NR | Con Fl | P | UNR |  | ***UNR*** | ***UNR*** |  |  | NR | ***Low*** | ***UNR*** |  |
|  | TUJ1 IHS |  |  |  | P | NR | Con Fl | P | UNR |  | ***UNR*** | ***UNR*** |  |  | NR | ***Low*** | ***UNR*** |  |
| Yamashita 2007 | Fas III (IHS) |  |  |  | P | NR | Con Fl | NR | UNR |  | ***UNR*** | ***UNR*** |  |  | NR | ***Low*** | ***UNR*** | No controls, unclear if consideration of focal plane |
|  | GFP PACT | D | no | NR |  |  | Con Fl | NR | UNR |  | ***UNR*** | ***UNR*** | Yes. | Appears to be visual inspection, which is quantified by cell number. | yes | ***Low*** | ***UNR*** | No controls, no genetic control, unclear if consideration of focal plane. Cellular location is accurate.  Assumes 40-50% of non-proximal labelled centrioles were assembled before GFP PACT expression. |
|  | Vasa (IHS) |  |  |  | P | NR | Con Fl | NR | UNR |  | ***UNR*** | ***UNR*** |  |  | NR | ***Low*** | ***UNR*** | No controls, unclear if consideration of focal plane |
| Rusan 2007 | Cnn-GFP | P | no | NR |  |  | Con Fl | P | UNR |  | ***UNR*** | ***UNR*** | NR | Asymmetry measure is unclear, appears to be visual inspection. | NR | ***Low*** | ***UNR*** | Lack of controls, unclear imaging techniques, no genetic manipulation control. Cellular location is accurate. Unclear if focal plane considered.  Asymmetry measure is unclear. |
| ***Centrosome (cilia)*** | | | | | | | | | | | | | | | | | | |
| Anderson 2009 | Centrin 2 GFP | P | yes | NR |  |  | FL | P | UNR |  | ***UNR*** | ***Low*** | Yes | Asymmetry measure is unclear, appears to be visual inspection. | yes | ***Low*** | ***UNR*** | Assumes that mitotic shake off produces only mitotic pairs (not two cells sticking together).  Cellular location is strong evidence of validity.  Asymmetry was also observed in untransfected NIH 3T3 cells and hTert-RPE1 human epithelial cells. No marker controls.  Unclear if optical plane considered |
|  | α tubulin IHS |  |  | NR | P | NR | FL | P | UNR |  | ***UNR*** | ***Low*** |  |  | yes | ***Low*** | ***UNR*** |  |
|  | α tubulin - myc | P | yes | yes |  |  | FL | P | UNR |  | ***UNR*** | ***Low*** |  |  | yes | ***Low*** | ***UNR*** |  |
| Piotrowska-Nitsche 2012 | Sstr3 GFP | P | yes | yes |  |  | Con Fl | D | NA |  | ***Low*** | ***UNR*** |  | Asymmetry measure is unclear, appears to be visual inspection. | NR | ***Low*** | ***UNR*** | Live cell imaging does not clearly reveal cilia, no marker controls.  untransfected cells also show asymmetry. |
|  | Sstr3 GFP virus | P | yes | yes |  |  | Con Fl | D | NA |  | ***UNR*** | ***UNR*** |  |  | NR | ***Low*** | ***UNR*** |  |
| ***Endoplasmic Reticulum*** | | | | | | | | | | | | | | | | | | |
| Dalton 2013 | DiI 18 |  |  |  | D | NR | Con Fl | P | yes |  | ***Low*** | ***UNR*** |  |  | NR | ***Low*** | ***UNR*** | lack of controls. |
| Smyth 2015 | Baz IHS |  |  |  | D | NR | Con Fl | P | yes |  | ***Low*** | ***UNR*** | Yes. | Total fluorescence within equally sized regions (approx. 2.25 mm in diameter) centred around each spindle pole was calculated. Background from identical regions was subtracted from each pole measurement to obtain corrected ER fluorescence intensities at each apical and basal pole. Total cellular ER fluorescence intensities were similarly calculated from the same images using regions that encompassed all of the ER | NR | ***UNR*** | ***UNR*** | Cellular localisation is relevant but no marker controls.  Shape of the ER envelope and by timing relative to anaphase onset, while apico-basal polarity was determined based on the size of the progeny cells following division. |
|  | Sec61 α GFP | P | no | NR |  |  | Con Fl | P | yes |  | ***Low*** | ***UNR*** |  |  | NR | ***Low*** | ***UNR*** |  |
| ***Endosome*** | | | | | | | | | | | | | | | | | | |
| Beckmann 2007 | CD53 |  |  |  | D | NR | FL | D | NA |  | ***Low*** | ***UNR*** |  | Asymmetry is unclear, appears to be visual inspection. | yes | ***Low*** | ***UNR*** | No controls.  Localised to vesicles (unclear if endosomes). Asymmetry measure is unclear. |
|  | CD63 |  |  |  | D. | NR | FL | D | NA |  | ***Low*** | ***UNR*** |  |  | yes | ***Low*** | ***UNR*** | No controls. Localised to vesicles (unclear if endosomes). Asymmetry measure is unclear. |
|  | CD71 |  |  |  | D | NR | FL | D | NA |  | ***Low*** | ***UNR*** |  |  | yes | ***Low*** | ***UNR*** | No controls. Localised to vesicles (unclear if endosomes). Asymmetry measure is unclear. |
| Coumailleau 2009 | FYVE GFP | P | no | NR |  |  | Con Fl | D | yes |  | ***UNR*** | ***UNR*** |  | For each time point, the sum of the total intensities measured for the 18 z sections was made to determine distribution of endosomes, and the ratio between “pIIb region” and “pIIa region” was plotted. Quantifications using Metamorph software. | NR | ***Low*** | ***UNR*** | No genetic controls or marker controls reported. No results for other models. Unclear marker validity |
|  | Pon RFP | P | no | NR |  |  | Con Fl | D | yes |  | ***UNR*** | ***UNR*** |  |  | yes | ***Low*** | ***UNR*** | No genetic controls or marker controls reported. No results for other models. Accepted marker and localisation |
|  | PtdIns(3)P GFP | P | no | NR |  |  | Con Fl | D | yes |  | ***UNR*** | ***UNR*** |  |  | NR | ***Low*** | ***UNR*** | No genetic controls or marker controls reported. No results for other models.  Marker could be any intracellular vesicle. |
|  | Rab5 GFP | P | no | NR |  |  | Con Fl | D | yes |  | ***UNR*** | ***UNR*** |  |  | NR | ***Low*** | ***UNR*** | No genetic controls or marker controls reported. No results for other models. Accepted marker |
|  | SARA GFP | P | no | NR |  |  | Con Fl | D | yes |  | ***UNR*** | ***UNR*** |  |  | yes | ***Low*** | ***UNR*** | No genetic controls or marker controls reported. No results for other models. Unclear marker validity |
| Emery 2005 | Pon RFP | P | no | NR |  |  | Con Fl | D | yes |  | ***UNR*** | ***UNR*** | NR |  | yes | ***Low*** | ***UNR*** | No genetic or marker controls |
|  | Rab11 GFP | P | yes | NR |  |  | Con Fl | D | yes |  | ***UNR*** | ***UNR*** | NR | Unclear asymmetry measure, appears to be visual inspection. Rab11-GFP intensity was calculated for all Z-planes using ImageJ. | yes | ***Low*** | ***UNR*** | No genetic or marker controls. Authors report that similar asymmetry can be seen using a Rab11 antibody indicating that the GFP fusion correctly reveals the localization of endogenous Rab11 |
|  | Rab5 GFP | P | no | NR |  |  | Con Fl | D | yes |  | ***UNR*** | ***UNR*** | NR | Unclear asymmetry measure, appears to be visual inspection. | yes | ***Low*** | ***UNR*** | No genetic or marker controls.  Accepted markers |
|  | Rab7 GFP | P | no | NR |  |  | Con Fl | D | yes |  | ***UNR*** | ***UNR*** | NR |  | yes | ***Low*** | ***UNR*** |  |
| Kressman 2015 | Rab11a YFP | P | no | NR |  |  | Con Fl | D | yes |  | ***UNR*** | ***UNR*** | NR | Sara- positive structures were segmented by manual thresholding of the CFP Sara (or Mib GFP) signal (set to encompass the smallest vesicle visible). Normalized intensities were then maximum projected along the z-axis in each cell. The ratio of Sara endosomes was calculated by dividing the highest normalized intensity between the two cells by the lowest. | NR | ***Low*** | ***UNR*** | Unclear if it does mark recycling endosomes and no marker or genetic controls. |
|  | Rab5c YFP | P | no | NR |  |  | Con Fl | D | yes |  | ***UNR*** | ***UNR*** | NR |  | NR | ***Low*** | ***UNR*** | No marker or genetic controls, unclear how marker -YFP generated. Accepted marker |
|  | Rab7 YFP | P | no | NR |  |  | Con Fl | D | yes |  | ***UNR*** | ***UNR*** | NR |  | NR | ***Low*** | ***UNR*** |  |
|  | Sara Venus | P | no | NR |  |  | Con Fl | D | yes |  | ***UNR*** | ***UNR*** | NR |  | yes | ***Low*** | ***High*** | Unclear that SARA marks early endosomes; Rab5c co-localises with SARA but doesn't show asymmetry, therefore something is incorrect. Authors state SARA co-localises with Rab 5c (fig S2b), however Rab5c does not show asymmetry therefore SARA cannot always co-localise with early endosomes. No genetic controls. Referenced positive control. |
| Loubery 2014 | Pon RFP | P | no | NR |  |  | Con Fl | D | yes |  | ***Low*** | ***UNR*** | NR | Total endosomal intensity in pIIa and the pIIb cells (IpIIa and IpIIb, respectively) was measured by integrating the intensity values in each slice of the first  z-stack after abscission, after having subtracted the background and thresholded the endosomes; the % of endosomes in the pIIa cell was computed as IpIIa / (IpIIa + IpIIb) | NR | ***Low*** | ***UNR*** | No genetic or marker controls. Accepted marker and localisation |
|  | Sara GFP | P | no | NR |  |  | Con Fl | D | yes |  | ***Low*** | ***UNR*** | NR |  | yes | ***Low*** | ***UNR*** | No genetic or marker controls |
| Montagne 2014 | PH3 IHS |  |  |  | D | NR | Con Fl | D | yes |  | ***Low*** | ***UNR*** |  | Unclear asymmetry measure, appears to be visual inspection. | NR | ***Low*** | ***UNR*** | No genetic or marker controls. Unclear if PH3 labels ISC? |
|  | Pon RFP | D | no | NR |  |  | Con Fl | D | yes |  | ***UNR*** | ***UNR*** |  |  | NR | ***Low*** | ***UNR*** | No genetic or marker controls. Accepted marker and localisation |
|  | Rab 11 GFP | D | no | NR |  |  | Con Fl | D | yes |  | ***UNR*** | ***UNR*** |  | Background signal was adjusted. Raw density in maternal cells was divided by the raw density in the daughter cells | NR | ***Low*** | ***UNR*** | No genetic or marker controls. Unclear marker. |
|  | Rab 7 GFP | D | no | NR |  |  | Con Fl | D | yes |  | ***UNR*** | ***UNR*** |  |  | NR | ***Low*** | ***UNR*** | No genetic or marker controls.  Accepted marker |
|  | SARA RFP | D | no | NR |  |  | Con Fl | D | yes |  | ***UNR*** | ***UNR*** |  |  | yes | ***Low*** | ***UNR*** | No genetic or marker controls. Unclear marker validation |
|  | αtubulin mcherry | D | no | NR |  |  | Con Fl | D | yes |  | ***UNR*** | ***UNR*** |  |  | NR | ***Low*** | ***UNR*** | No genetic or marker controls. Accepted marker. |
| ***Golgi*** | | | | | | | | | | | | | | | | | | |
| Katajisto 2015 | 1,4-galactosyltransferase (81 N-term aa) paGFP | P | no | NR |  |  | Con Fl | P | yes |  | ***UNR*** | ***UNR*** |  | For quantitation of asymmetric divisions, acquired images were merged to a composite image(ImageJ). Intensity measurements were made from frames one-hour before and after division. Fluorescence was reported as a percentage of the maternal cell | NR | ***Low*** | ***UNR*** | No controls, no second markers, no gene manipulation controls. |
| ***Lysosome*** | | | | | | | | | | | | | | | | | | |
| Katajisto 2015 | lamp2 pa GFP | P | no | NR |  |  | Con Fl | P | yes |  | ***UNR*** | ***UNR*** | yes | For quantitation of asymmetric divisions, acquired images were merged to a composite image(ImageJ). Intensity measurements were made from frames one-hour before and after division. Fluorescence was reported as a percentage of the maternal cell | NR | ***UNR*** | ***UNR*** | No controls, no second markers, no gene manipulation controls.  Assumes mammosphere formation is consistent with stem cell properties |
| ***Midbody*** | | | | | | | | | | | | | | | | | | |
| Goss 2008 | αMKLP1 |  |  |  | P | NR | Con Fl | L | yes |  | ***Low*** | ***Low*** |  | Unclear, but asymmetry appears to be a qualitative visual inspection (yes or no), which is subsequently quantified for cell number. | NR | ***Low*** | ***UNR*** | Lack of controls and unclear how cells with midbody were counted, number of cells with a small spot? Were all these analysed by confocal? Cellular localisation is consistent with mid body. Result repeated with Hela cell line. |
| Kuo 2014 | CETN1-GFP | P | no | NR |  |  |  |  |  |  | ***UNR*** | ***Low*** | Yes | Unclear, but asymmetry appears to be a qualitative visual inspection (yes or no), which is subsequently quantified for cell number. | NR | ***UNR*** | ***UNR*** | Lack of genetic and marker controls; Assumes centrin marks the maternal centriole.  Result repeated with Hela cell line; U2OS cell line. |
|  | MKLP1 IHS |  |  |  | P | NR | FL | P | NA |  | ***Low*** | ***Low*** |  |  | NR | ***Low*** | ***UNR*** | No controls. Result repeated with Hela cervix adenocarcinoma cell line; U2OS ostosarcoma cell line |
|  | α tubulin IHS |  |  |  | P | NR | FL | P | NA |  | ***Low*** | ***Low*** |  |  | NR | ***Low*** | ***UNR*** | No controls. Accepted marker. Result repeated with Hela and U2OS cell lines. |
| Saltzmann 2013 | Add IHS |  |  |  | D | NR | Con Fl | L | UNR |  | ***UNR*** | ***UNR*** |  | Unclear, but asymmetry appears to be a qualitative visual inspection (yes or no), which is subsequently quantified for cell number. | NR | ***Low*** | ***UNR*** | Poor stain and lack of controls  Unclear if focal plane considered. |
|  | Fas III  IHS |  |  |  | D | NR | Con Fl | L | UNR |  | ***UNR*** | ***UNR*** |  |  | NR | ***Low*** | ***UNR*** | No controls, unclear if focal plane considered. Unclear marker validity. |
|  | GFP Pavarotti | P | no | NR |  |  | Con Fl | L | UNR |  | ***UNR*** | ***UNR*** |  |  | NR | ***Low*** | ***UNR*** | No marker or genetic controls. Cellular location is good validity. Unclear if focal plane considered. |
|  | Spd-2  IHS |  |  |  | D | NR | Con Fl | L | UNR |  | ***UNR*** | ***UNR*** |  |  | NR | ***Low*** | ***UNR*** | Poor stain and lack of controls. Cellular location is good validity. Unclear if focal plane considered. |
|  | Vasa  IHS |  |  |  | D | UNR | Con Fl | L | UNR |  | ***UNR*** | ***UNR*** |  |  | NR | ***Low*** | ***UNR*** | Unclear which antibody was used. Unclear if focal plane considered. |
|  | γ-tubulin  IHS |  |  |  | NR | NR | Con Fl | L | UNR |  | ***UNR*** | ***UNR*** |  |  | NR | ***Low*** | ***UNR*** | Due to cellular localisation. Unclear immunostaining method (primary antibody).  Unclear if focal plane considered. |
| ***Mitochondria*** | | | | | | | | | | | | | | | | | | |
| Dalton 2013 | Cyto-chrome c  IHS |  |  |  | P | NR | Con Fl | P | yes |  | ***Low*** | ***UNR*** |  | The asymmetry score was calculated where the chromosomes divided in the plane of imaging. The metaphase plate or the central point between the dividing chromosomes was defined as the mid-point and the average fluorescence of mitochondria either side of this point on the line scan was measured and a ratio calculated. | NR | ***Low*** | ***UNR*** | Lack of marker controls. |
|  | mito-GFP | P | no | NR |  |  | Con Fl | P | yes |  | ***Low*** | ***UNR*** |  |  | NR | ***Low*** | ***UNR*** | Lack of genetic and marker controls. |
|  | TOM20  IHS |  |  |  | P | NR | Con Fl | P | yes |  | ***Low*** | ***UNR*** |  |  | NR | ***Low*** | ***UNR*** | Lack of marker controls. |
| Katajisto 2015 | Omp25 paGFP | P | no | NR |  |  | Con Fl | P | yes |  | ***UNR*** | ***Low*** |  | For quantitation of asymmetric divisions, acquired images were merged to a composite image(ImageJ). Intensity measurements were made from frames one-hour before and after division. Fluorescence was reported as a percentage of the maternal cell | NR | ***Low*** | ***UNR*** | No marker or genetic controls.  Cellular localisation is consistent with use of mito tracker.  Result repeated with FL1 and Fl2 mammary epithelial cell lines. |
| Rivolta 2002 | Mab48  IHS |  |  |  | P | NR | NR | NR | UNR |  | ***UNR*** | ***Low*** | Yes | Cytoplasm was divided in two regions based on the position of the nucleus and a cell scored as ‘asymmetric’ if more than 90% of one region was devoid of labelling. | yes | ***Low*** | ***UNR*** | No marker controls, imaging methods are unclear and the pictures are over exposed. MitoTracker Red CMXRos was used as a second marker for mitochondria and labelling was coincident with mab48.  Result repeated with 2 other cell lines. Assumes ‘asymmetric’ if more than 90% of one region was devoid of labelling and assumes all cells were cycling for this to be asymmetric inheritance |
| ***P granule*** | | | | | | | | | | | | | | | | | | |
| Gallo 2010 | PGL1 GFP | P | no | NR |  |  | Con Fl | P | yes |  | ***UNR*** | ***UNR*** |  | Sum fluorescence intensities were measured in the P blastomere and in an identically-sized region positioned over the somatic sister. Fold enrichment was calculated as the average ratio of P cell:soma. | yes | ***Low*** | ***Low*** | Localisation is good, Similar results with GFP fusions to two other P granule proteins PGL-3 and GLH-1. No negative controls.  Asymmetry measure is clear. |
| Rose 1998 | P granule antibody OIC1D4 or K76 |  |  |  | L | NR | Con Fl | L | no |  | ***UNR*** | ***UNR*** |  | Asymmetry measure is unclear, appears to be visual inspection | NR | ***Low*** | ***UNR*** | No negative controls, unclear microscopy methods, unclear which antibody used. Asymmetry measure is unclear. |
| Boyd 1996 | P granule antibody OIC1D4 |  |  |  | D | NR | NR | NR | no |  | ***UNR*** | ***UNR*** | NR | Asymmetry measure is unclear, appears to be visual inspection | NR | ***Low*** | ***UNR*** | Unclear image analysis, no controls, marker is referenced |
| Pang 2004 | K76  IHS |  |  |  | NR | NR | Con Fl | NR | UNR |  | ***UNR*** | ***UNR*** |  | Unclear asymmetry measure, appears to be visual inspection. | NR | ***Low*** | ***UNR*** | Limited experimental details. No controls. K76 is a known marker of p granules and the staining looks consistent  Unclear if focal plane considered. |
| ***Proteosome*** | | | | | | | | | | | | | | | | | | |
| Chang 2011 | proteosome 20s alpha 1 |  |  |  | Ref | NR | Con Fl | D | yes |  | ***Low*** | ***Low*** | NR | Receptor enrichment in one hemisphere or in one nascent daughter cell greater than 1.5-fold compared to the other hemisphere or daughter cell was considered polarized | NR | ***Low*** | ***UNR*** | No negative controls reported.  Authors use further antibodies to distinct proteosomal epitopes and also find asymmetry. Results repeated with two T cells models.  Limited methodology |
| Ogrodnik 2014 | mito-dendra 2 | P | no | NR |  |  | Con Fl | P | yes |  | ***UNR*** | ***UNR*** |  | Unclear asymmetry measure, appears to be visual inspection. | NR | ***Low*** | ***UNR*** | No genetic or marker controls, unclear which cell line used. Accepted marker and location strong evidence |
|  | pDendra2-VHL | P | no | yes |  |  | Con Fl | P | yes |  | ***UNR*** | ***Low*** |  |  |  | ***UNR*** | ***UNR*** | No genetic or marker controls, unclear which cell line used. stated in text that untransfected cells acted as a control but did not present evidence Co-localization with other markers. Authors show that JUNQ co-localises to proteosome activity in Fig1H and Fig1I.  Result repeated in yeast. |
| ***Spectrosome/ fusome*** | | | | | | | | | | | | | | | | | | |
| de Cuevas 1998 | Anillin  IHS |  |  |  | P | NR | Con Fl | L | yes |  | ***Low*** | ***UNR*** |  |  | NR | ***Low*** | ***UNR*** | No controls. Celllular localisation is good evidence for validity  Limited methodology.  Assumes any single cell separated from the base of the terminal filament by at least one other cell was considered a cystoblast. |
|  | hts protein  IHS |  |  |  | Ref | NR | Con Fl | L | yes |  | ***Low*** | ***UNR*** | Yes | Asymmetry measure is unclear but appears to be quantified by visual inspection | NR | ***UNR*** | ***UNR*** |  |
| Lin 1995 | α spectrin  IHS |  |  |  | Ref | NR | Con Fl | L | yes |  | ***Low*** | ***UNR*** | Yes | Asymmetry measure is unclear but appears to be quantified by visual inspection | NR | ***UNR*** | ***UNR*** | No controls. Cellular localisation is good evidence for validity  Limited methodology. Cytoblasts were assumed to be the mitotic cells posterior to the presumptive stem cells (which were mitotic cells adjacent to the terminal filaments). |
|  | α tubulin IHS |  |  |  | Ref | NR | Con Fl | L | yes |  | ***Low*** | ***UNR*** | Yes |  | NR | ***UNR*** | ***UNR*** |  |
| UNR= unclear or not reported; NR = not reported; U = unclear; NA = not applicable; Ref = referenced;L= likely; D = detailed; P = partial; pa = photoactivatable; GFP = green fluorescent protein; RFP = red fluorescent protein; YFP = yellow fluorescent protein; Cnb = centrobin; Cnn = centrosomin; IHS = immune; Con Fl – confocal fluorescent microscopy; Fl = fluorescent.  Maternal organelle = original organelle, whilst daughter = newly synthesised organelle. | | | | | | | | | | | | | | | | | | |
