# Supplementary material for: A systematic review of the asymmetric inheritance of cellular organelles in eukaryotes: A critique of basic science validity and imprecision
Source: PLoS One. 2017 May 31;12(5):e0178645. doi: 10.1371/journal.pone.0178645 (PMC5451095; doi:10.1371/journal.pone.0178645)
Supplement: S5 Table — (DOCX) [file pone.0178645.s007.docx]

**S5 Table. Risk of bias (SYRCLE)**

| First Author Surname and Year | 1. allocation seq | 2. baseline/  confounders | 3. allocation conceal | 4. random housing | 5. investigators blinded | 6. animals assessed at random | 7. blinding of outcome assessor | 8. incomplete outcome data | 9. selective outcome reporting | 10. other bias |
| --- | --- | --- | --- | --- | --- | --- | --- | --- | --- | --- |
| Anderson 2009 | UNR | UNR | UNR | NA | UNR | NA | UNR | UNR | UNR | UNR |
| Beckmann 2007 | UNR | UNR | UNR | UNR | UNR | NA | UNR | UNR | UNR | UNR |
| Boyd 1996 | UNR | UNR | UNR | UNR | UNR | UNR | UNR | UNR | UNR | UNR |
| Chang 2011 | UNR | UNR | UNR | UNR | UNR | UNR | UNR | UNR | UNR | UNR |
| Conduit 2010 | UNR | UNR | UNR | UNR | UNR | UNR | UNR | UNR | UNR | UNR |
| Coumailleau 2009 | UNR | UNR | NA | NA | UNR | UNR | UNR | UNR | UNR | UNR |
| Dalton 2013 | UNR | UNR | UNR | UNR | UNR | UNR | UNR | UNR | UNR | UNR |
| de Cuevas 1998 | UNR | UNR | UNR | UNR | UNR | UNR | UNR | UNR | UNR | UNR |
| Emery 2005 | UNR | UNR | UNR | UNR | UNR | UNR | UNR | UNR | UNR | UNR |
| Gallo 2010 | UNR | UNR | UNR | UNR | UNR | UNR | UNR | UNR | UNR | UNR |
| Goss 2008 | NA | NA | UNR | NA | UNR | NA | UNR | UNR | UNR | UNR |
| Holy 1991 | UNR | UNR | UNR | UNR | UNR | UNR | UNR | UNR | UNR | UNR |
| Januschke 2011 | UNR | UNR | UNR | UNR | UNR | UNR | UNR | UNR | UNR | UNR |
| Katajisto 2015 | UNR | UNR | UNR | NA | UNR | NA | UNR | UNR | UNR | UNR |
| Kressmann 2015 | UNR | UNR | UNR | NA | UNR | UNR | UNR | UNR | UNR | UNR |
| Kuo 2014 | UNR | NA | UNR | NA | UNR | NA | UNR | UNR | UNR | UNR |
| Lin 1995 | UNR | UNR | UNR | UNR | UNR | UNR | UNR | UNR | UNR | UNR |
| Loubery 2014 | UNR | UNR | UNR | UNR | UNR | UNR | UNR | UNR | UNR | UNR |
| Montagne 2014 | UNR | UNR | UNR | UNR | UNR | UNR | UNR | UNR | UNR | UNR |
| Ogrodnik 2014 | UNR | UNR | UNR | UNR | UNR | UNR | UNR | UNR | UNR | UNR |
| Pang 2004 | UNR | UNR | UNR | NA | UNR | UNR | UNR | UNR | UNR | UNR |
| Piotrowska-Nitsche 2012 | UNR | UNR | UNR | UNR | UNR | UNR | UNR | UNR | UNR | UNR |
| Rivolta 2002 | UNR | UNR | UNR | UNR | UNR | UNR | UNR | UNR | UNR | UNR |
| Rose 1998 | UNR | UNR | UNR | UNR | UNR | UNR | UNR | UNR | UNR | UNR |
| Rusan 2007 | UNR | UNR | UNR | UNR | UNR | UNR | UNR | UNR | UNR | UNR |
| Salzmann 2013 | UNR | UNR | UNR | UNR | UNR | UNR | UNR | UNR | UNR | UNR |
| Shimizu 1996 | UNR | UNR | UNR | UNR | UNR | UNR | UNR | UNR | UNR | UNR |
| Smyth 2015 | UNR | UNR | UNR | UNR | UNR | UNR | UNR | UNR | UNR | UNR |
| Tamura 2001 | UNR | UNR | UNR | UNR | UNR | UNR | UNR | UNR | UNR | UNR |
| Wang 2009 | UNR | UNR | UNR | UNR | UNR | UNR | UNR | UNR | UNR | UNR |
| Yamashita 2007 | UNR | UNR | UNR | UNR | UNR | UNR | UNR | UNR | UNR | UNR |
| UNR =unclear or not reported; NA= not applicable | | | | | | | | | | |
