# Supplementary material for: A systematic review of the asymmetric inheritance of cellular organelles in eukaryotes: A critique of basic science validity and imprecision
Source: PLoS One. 2017 May 31;12(5):e0178645. doi: 10.1371/journal.pone.0178645 (PMC5451095; doi:10.1371/journal.pone.0178645)
Supplement: S6 Table — (DOC) [file pone.0178645.s008.doc]

**S6 Table. Validity assessments and adherence to reporting guidelines in systematic reviews of cellular r**esearch

| **Systematic review** | **Adherence to reporting guidelines?** | **Formal risk of bias assessment** | **Did authors assess model validity to establish how reliable the results were?** | **Did the authors investigate marker or outcome validity to establish how reliable the results were?** | **Did the authors consider the imprecision of the results?** | **Reporting of technical repeats** | **Reporting of inter-assay repeats** | **Reporting of variability** |
| --- | --- | --- | --- | --- | --- | --- | --- | --- |
| Napotnik 2016[1] | No | Cochrane collaboration risk of bias (majority of studies rated unclear) | No | Authors provided some discussion of experimental validity including compliance rate of evaluations. | Incomplete outcome data only | No | No | No |
| Hooper 2009[2] | No | No | No | No | No | No | No | No |
| Calton 2015[3] | PRISMA | No | No | No | No | No | No | No |
| Pavan 2015[4] | PRISMA | GRADE (unclear how the judgements were applied to basic research). Also, evaluated the evidence based on: (1) showing a potential effect; (2) inconclusive and (3) not supportive. | Downgraded one study for using only one cell line. | Unclear | Unclear | No | No | No |
| Bus 2012[5] | No | No | Limitations of different cell lines discussed | Discussion of outcomes | No | No | No | No |
| Mafi 2011[6] | No | No | No | No | No | No | No | No |
| Shanbhag 2016[7] | PRISMA and ARRIVE | SYRCLE (overall, the included studies were of unclear to high ROB and moderate reporting quality). | No for endothelial cells, osteoblasts or stem cells. Details of host animal provided | No | Yes | Yes | Yes | Yes |
| Bastami 2016[8] | Presentation of PRISMA flow diagram and clearly presents methods and results in line with PRISMA. | No | Partial. Description of model details and how they were reprogrammed or induced | No | No | No | No | No |
| Ramamoorthi 2015[9] | ARRIVE and CONSORT | Cochrane (most studies reported no sample size calculation, allocation concealment, randomisation or blinding) | Yes, for ARRIVE questions relating to methodology reporting but no validation of whether the models could represent functional stem cells. | No judgement on the validity of chosen outcomes. | Sample size. Yes, as described in ARRIVE and CONSORT, but not fed into which are the most reliable results. | Yes | Yes | Descriptive outcomes |
| Wang 2010[10] | No | No | No | No | No | Yes | No | No |
| Gudbergsson 2016[11] | No, but presentation of PRISMA flow diagram | No | Considered method of isolation in detail. The influence of cell type and confluency was considered. | Quantification method of extra-cellular vesicle yield was considered. Impact of purification methods on yield was considered. Common markers found on exosomes as a way of determining false positives. | Not for individual studies | No | No | Box -Whisker plots |
| Harkin 2016[12] | No | No | Yes (origin of cells tracked). | Yes (appropriate marker used, how was marker validated and controls) | No | No | No | No |
| Xiao 2011[13] | No | Based on authors grading: A Systematic reviews/ meta-analyses of studies in vitro, B With comparable baseline, C Baseline unknown, D No comparable baseline. All were assigned grade B. | No | No. Controls reported for culture conditions but not for the markers. | No | No | No | No |
| Hynes 2016[14] | PRISMA | Based on randomization, blinding, allocation concealment, incomplete data and selective outcome reporting. No evidence was found for the latter three domains. | No | No | Sample size was considered, only 1/30 studies reported a calculation. | No | No | No |
| Korpershoek 2017[15] | PRISMA | No | Described stem cell sources, queried the cell type required for regeneration had not been defined precisely. No functional validation of stem cell properties. | No | Number of cells required for regeneration | No | No | No |
| Rahman 2016[16] | No | A modified Quality Assessment Tool for Studies with Diverse Designs (Sirriyeh et al., 2012); average score range 13 -35 | Passage number and protein markers of endothelial cells. | No | No | No | No | No |

1. Batista Napotnik T, Reberšek M, Vernier PT, Mali B, Miklavčič D. Effects of high voltage nanosecond electric pulses on eucaryotic cells (in vitro): A systematic review. Bioelectrochemistry. 2016;110: 1–12. doi:10.1016/j.bioelechem.2016.02.011

2. Hooper SJ, Wilson MJ, Crean SJ. Exploring the link between microorganisms and oral cancer: A systematic review of the literature. Myers JN, editor. Head & Neck. 2009;31: 1228–1239. doi:10.1002/hed.21140

3. Calton EK, Keane KN, Newsholme P, Soares MJ. The impact of Vitamin D levels on inflammatory status: A systematic review of immune cell studies. PLoS ONE. 2015;10: 1–12. doi:10.1371/journal.pone.0141770

4. Pavan LM, Rego DF, Elias ST, De Luca Canto G, Guerra EN. In vitro Anti-Tumor Effects of Statins on Head and Neck Squamous Cell Carcinoma: A Systematic Review. PLoS One. 2015;10: e0130476. doi:10.1371/journal.pone.0130476

5. Bus P, Siersema PD, Van Baal JWPM. Cell culture models for studying the development of Barrett’s esophagus: A systematic review. Cellular Oncology. 2012;35: 149–161. doi:10.1007/s13402-012-0076-6

6. P M, S H, R M, M G, W S K. Adult mesenchymal stem cells and cell surface characterization - a systematic review of the literature. The open orthopaedics journal. 2011;5: 253–60. doi:10.2174/1874325001105010253

7. Shanbhag S, Pandis N, Mustafa K, Nyengaard JR, Stavropoulos A. Cell Cotransplantation Strategies for Vascularized Craniofacial Bone Tissue Engineering: A Systematic Review and Meta-Analysis of Preclinical *In Vivo* Studies. Tissue Engineering Part B: Reviews. 2016;23: ten.teb.2016.0283. doi:10.1089/ten.teb.2016.0283

8. Bastami F, Nazeman P, Moslemi H, Rezai Rad M, Sharifi K, Khojasteh A. Induced pluripotent stem cells as a new getaway for bone tissue engineering: A systematic review. Cell Proliferation. 2016; 1–29. doi:10.1111/cpr.12321

9. Ramamoorthi M, Bakkar M, Jordan J, Tran SD. Osteogenic Potential of Dental Mesenchymal Stem Cells in Preclinical Studies: A Systematic Review Using Modified ARRIVE and CONSORT Guidelines. Stem cells international. 2015;2015: 378368. doi:10.1155/2015/378368

10. Wang L, Xie Y, Zhu L-J, Chang T-T, Mao Y-Q, Li J. An association between immunosenescence and CD4(+)CD25(+) regulatory T cells: a systematic review. Biomedical and environmental sciences : BES. 2010;23: 327–332. doi:10.1016/S0895-3988(10)60072-4

11. Gudbergsson JM, Johnsen KB, Skov MN, Duroux M. Systematic review of factors influencing extracellular vesicle yield from cell cultures. Cytotechnology. Springer Netherlands; 2016;68: 579–592. doi:10.1007/s10616-015-9913-6

12. Harkin DG, Foyn L, Bray LJ, Sutherland AJ, Li FJ, Cronin BG. Concise reviews: can mesenchymal stromal cells differentiate into corneal cells? A systematic review of published data. Stem cells (Dayton, Ohio). 2015;33: 785–791. doi:10.1002/stem.1895

13. Xiao Z, Li C, Shan J, Luo L, Feng L, Lu J, et al. Mechanisms of renal cell apoptosis induced by cyclosporine A: A systematic review of in vitro studies. American Journal of Nephrology. 2011;33: 558–566. doi:10.1159/000328584

14. Hynes K, Bright R, Proudman S, Haynes D, Gronthos S, Bartold M. Immunomodulatory properties of mesenchymal stem cell in experimental arthritis in rat and mouse models: A systematic review. Seminars in Arthritis and Rheumatism. Elsevier; 2016;46: 1–19. doi:10.1016/j.semarthrit.2016.02.008

15. Korpershoek J V., de Windt TS, Hagmeijer MH, Vonk LA, Saris DBF. Cell-Based Meniscus Repair and Regeneration: At the Brink of Clinical Translation? Orthopaedic Journal of Sports Medicine. 2017;5: 232596711769013. doi:10.1177/2325967117690131

16. Rahman NA, Rasil ANHM, Meyding-Lamade U, Craemer EM, Diah S, Tuah AA, et al. Immortalized endothelial cell lines for in vitro blood-brain barrier models: A systematic review. Brain research. 2016;1642: 532–545. doi:10.1016/j.brainres.2016.04.024
